# Supplementary material for: Identification and Structural Analysis of Spirostanol Saponin from Yucca schidigera by Integrating Silica Gel Column Chromatography and Liquid Chromatography/Mass Spectrometry Analysis
Source: Molecules. 2020 Aug 24;25(17):3848. doi: 10.3390/molecules25173848 (PMC7504267; doi:10.3390/molecules25173848)
Supplement: Supplementary file 1 [file molecules-25-03848-s001.pdf]

Supporting information

Identification and structural analysis of spirostanol saponin from *Yucca schidigera* by integrating silica gel column chromatography and liquid chromatography/mass spectrometry analysis

Jingya Ruan [1,†], Lu Qu [1,3,†], Wei Zhao [1], Chang Gao [1], Peijian Huang [1], Dandan Zheng [2], Lifeng Han [2], Haiyang Yu [2], Zixin Zhang [2], Yi Zhang [1,2,*] and Tao Wang [1,2,*]

1 Tianjin Key Laboratory of TCM Chemistry and Analysis, Tianjin University of Traditional Chinese Medicine, 10 Poyanghu Road, West Area, Tuanbo New Town, Jinghai District, 301617 Tianjin, China; Ruanjy19930919@163.com (J.R.); qululuhan88@163.com (L.Q.); zhaowei126123@126.com (W.Z.); GC087159@163.com (C.G.); hpjforever@sina.com (P.H.);

2 Institute of TCM, Tianjin University of Traditional Chinese Medicine, 10 Poyanghu Road, West Area, Tuanbo New Town, Jinghai District, 301617 Tianjin, China; zhengdd1027@163.com (D.Z.); hanlifeng_1@sohu.com (L.H.); hyyu@tjutcm.edu.cn (H.Y.); zixinzhang0115@163.com (Z.Z.);

3 Yunnan University of Traditional Chinese Medicine, 1076 Yuhua Road, Chenggong District ,650500, Kunming, China

* Correspondence: zhwwxzh@tjutcm.edu.cn (Y.Z.); wangtao@tjutcm.edu.cn (T.W.); Tel./Fax: +86-22-5959-6168 (T.W.)

† These authors contributed equally to this manuscript.

Figure S1. BPC chromatograms of YS separated on different columns, Columns: BEH C18, HSS C18, C18 and T3 4
Figure S2. BPC chromatograms of YS separated on T3 column in different solvent systems...... 5
Figure S3. BPC chromatograms of YS separated on T3 column with different concentrations of FA 6
Figure S4. BPC chromatograms of YS separated on T3 column at different temperature............ 7
Figure S5. BPC chromatograms of YS, YSESs 6–9 and YSEMs 7–9 by OC-MS in positive ion 8
Figure S6. EIC of *m/z* 741.4420 from YS, YSM 7–9 and YSS 6.. 9
Figure S7. EIC of *m/z* 755.4223 from YSS 6 and YS.. 10
Figure S8. The MS/MS spectrum of peak **10**... 11
Figure S9. The MS/MS spectrum of peak **14**... 12
Figure S10. The MS/MS spectrum of peak **15**... 13
Figure S11. The MS/MS spectrum of peak **27**... 14
Figure S12. The MS/MS spectrum of peak **34**... 15
Figure S13. The MS/MS spectrum of peak **36**... 19
Figure S17. The MS/MS spectrum of peak **51**... 20
Figure S18. ^{1}H NMR (500MHz, C_5D_5N) spectrum of compound **10**.................................... 21
Figure S19. ^{13}C NMR (125MHz, C_5D_5N) spectrum of compound **10**.................................. 21
Figure S20. The DEPT 135 (C_5D_5N) spectrum of compound **10**.. 22
Figure S21. The ^{1}H ^{1}H COSY (C_5D_5N) spectrum of compound **10**.................................... 22
Figure S22. The HSQC (C_5D_5N) spectrum of compound **10**.. 23
Figure S23. The HMBC (C_5D_5N) spectrum of compound **10**... 23
Figure S24. The ESI-Q-Orbitrap MS spectrum of compound **10**.. 24
Figure S25. ^{1}H NMR (500MHz, C_5D_5N) spectrum of compound **14/15**............................... 25
Figure S26. ^{13}C NMR (125MHz, C_5D_5N) spectrum of compound **14/15**............................. 25
Figure S27. The ^{1}H ^{1}H COSY (C_5D_5N) spectrum of compound **14/15**............................... 26
Figure S28. The HSQC (C_5D_5N) spectrum of compound **14/15**... 26
Figure S29. The HMBC (C_5D_5N) spectrum of compound **14/15**... 27
Figure S30. The ESI-Q-Orbitrap MS spectrum of compound **14/15**... 27
Figure S31. ^{1}H NMR (500MHz, C_5D_5N) spectrum of compound **27**.................................... 28
Figure S32. ^{13}C NMR (125MHz, C_5D_5N) spectrum of compound **27**.................................. 28
Figure S33. The DEPT 135 (C_5D_5N) spectrum of compound **27**.. 29
Figure S34. The ^{1}H ^{1}H COSY (C_5D_5N) spectrum of compound **27**.................................... 29
Figure S35. The HSQC (C_5D_5N) spectrum of compound **27**.. 30
Figure S36. The HMBC (C_5D_5N) spectrum of compound **27**... 30
Figure S37. The HSQC-TOCSY (C_5D_5N) spectrum of compound **27**...................................... 31
Figure S38. The ESI-Q-Orbitrap MS spectrum of compound **27**.. 31
Figure S39. ^{1}H NMR (500MHz, C_5D_5N) spectrum of compound **34/36**............................... 32
Figure S40. ^{13}C NMR (125MHz, C_5D_5N) spectrum of compound **34/36**............................. 32
Figure S41. The DEPT 135 (C_5D_5N) spectrum of compound **34/36**....................................... 33
Figure S42. The ^{1}H ^{1}H COSY (C_5D_5N) spectrum of compound **34/36**............................... 33
Figure S43. The HSQC (C_5D_5N) spectrum of compound **34/36**... 34
Figure S44. The HMBC (C_5D_5N) spectrum of compound **34/36**.. 34
Figure S45. The ESI-Q-Orbitrap MS spectrum of compound **34/36**... 35
Figure S46. ^{1}H NMR (500MHz, C_5D_5N) spectrum of compound **38**.................................... 36
Figure S47. ^{13}C NMR (125MHz, C_5D_5N) spectrum of compound **38**.................................. 36
Figure S48. The DEPT 135 (C_5D_5N) spectrum of compound **38**.. 37
Figure S49. The ^{1}H ^{1}H COSY (C_5D_5N) spectrum of compound **38**.................................... 37
Figure S50. The HSQC (C_5D_5N) spectrum of compound **38**.. 38
Figure S51. The HMBC (C_5D_5N) spectrum of compound **38**... 38
Figure S52. The HSQC-TOCSY (C_5D_5N) spectrum of compound **38**...................................... 39
Figure S53. The ESI-Q-Orbitrap MS spectrum of compound **38**.. 39

Figure S54. ^{1}H NMR (500MHz, C_5D_5N) spectrum of compound **40/41**...................................... 40
Figure S55. ^{13}C NMR (125MHz, C_5D_5N) spectrum of compound **40/41**.................................... 40
Figure S56. The DEPT 135 (C_5D_5N) spectrum of compound **40/41**... 41
Figure S57. The ^{1}H ^{1}H COSY (C_5D_5N) spectrum of compound **40/41**....................................... 41
Figure S58. The HSQC (C_5D_5N) spectrum of compound **40/41**... 42
Figure S59. The HMBC (C_5D_5N) spectrum of compound **40/41**... 42
Figure S60. The ESI-Q-Orbitrap MS spectrum of compound **40/41**... 43
Figure S61. ^{1}H NMR (500MHz, C_5D_5N) spectrum of compound **51**.. 44
Figure S62. ^{13}C NMR (125MHz, C_5D_5N) spectrum of compound **51**.. 44
Figure S63. The DEPT 135 (C_5D_5N) spectrum of compound **51**... 45
Figure S64. The ^{1}H ^{1}H COSY (C_5D_5N) spectrum of compound **51**... 45
Figure S65. The HSQC (C_5D_5N) spectrum of compound **51**.. 46
Figure S66. The HMBC (C_5D_5N) spectrum of compound **51**.. 46
Figure S67. The ESI-Q-Orbitrap MS spectrum of compound **51**... 47
Table S1. ^{1}H and ^{13}C NMR data for compound **10** in C_5D_5N... 48
Table S2a. ^{1}H and ^{13}C NMR data of C-1–15, 1'–6', 1''–6'', and 1'''–6'''for compound **14/15** in C_5D_5N... 49
Table S2b. ^{1}H and ^{13}C NMR data of C-16–27 for compound **14/15** in C_5D_5N............................. 49
Table S3. ^{1}H and ^{13}C NMR data for compound **27** in C_5D_5N.. 50
Table S4a. ^{1}H and ^{13}C NMR data of C-1–15, 1'–6', and 1''–5'' for compound **34/36** in C_5D_5N.... 51
Table S4b. ^{1}H and ^{13}C NMR data of C-16–27 for compound **34/36** in C_5D_5N........................... 51
Table S5. ^{1}H and ^{13}C NMR data for compound **38** in C_5D_5N.. 52
Table S6a. ^{1}H and ^{13}C NMR data of C-1–15 and C-1'–6' for compound **40/41** in C_5D_5N.... 53
Table S6b. ^{1}H and ^{13}C NMR data of C-16–27 for compound **40/41** in C_5D_5N............................ 53
Table S7. ^{1}H and ^{13}C NMR data for compound **51** in C_5D_5N.. 54
Table S8. The characteristic types of substituted glycosyl groups.. 55
Table S9. The characteristic fragment ions of seven aglycone moieties..................................... 56
Table S10. The chromatographic elution order of spirostanol saponins from YSSs..................... 57

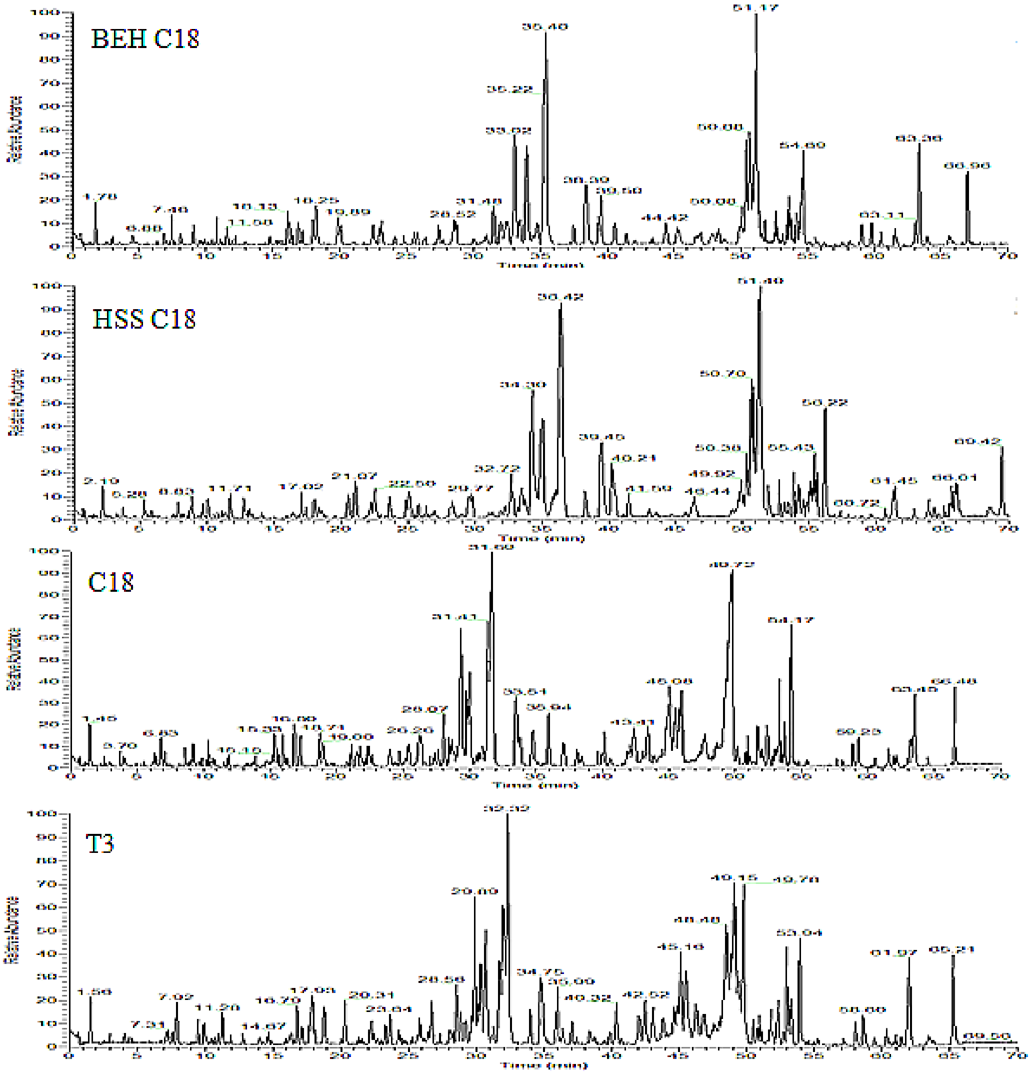

Figure S1. BPC for YS separated on different columns: BEH C18, HSS C18, C18 and T3. Column: Waters ACQUITY UPLC® BEH C_{18} (1.7 μm, 2.1 × 100 mm), Waters ACQUITY UPLC® HSS C_{18} (1.8 μm, 2.1 × 100 mm), Waters ACQUITY UPLC® C_{18} (1.8 μm, 2.1 × 100 mm) and Waters ACQUITY UPLC® T_3 (1.8 μm, 2.1 × 100 mm). Mobile phase: H_2O (A) and FA-MeOH-ACN (0.1:50:50, v/v/v) (B). Gradient program: 0-15 min, 12-24% B; 15-30 min, 24-32% B; 30-42 min, 32-40% B; 42-47 min, 40-60% B; 47-65 min, 60-95% B. Flow rate: 0.3 mL/min. Column temperature: 30°C. Injection volume: 3 μL. ESI-Q-Orbitrap MS mode: positive ion mode.

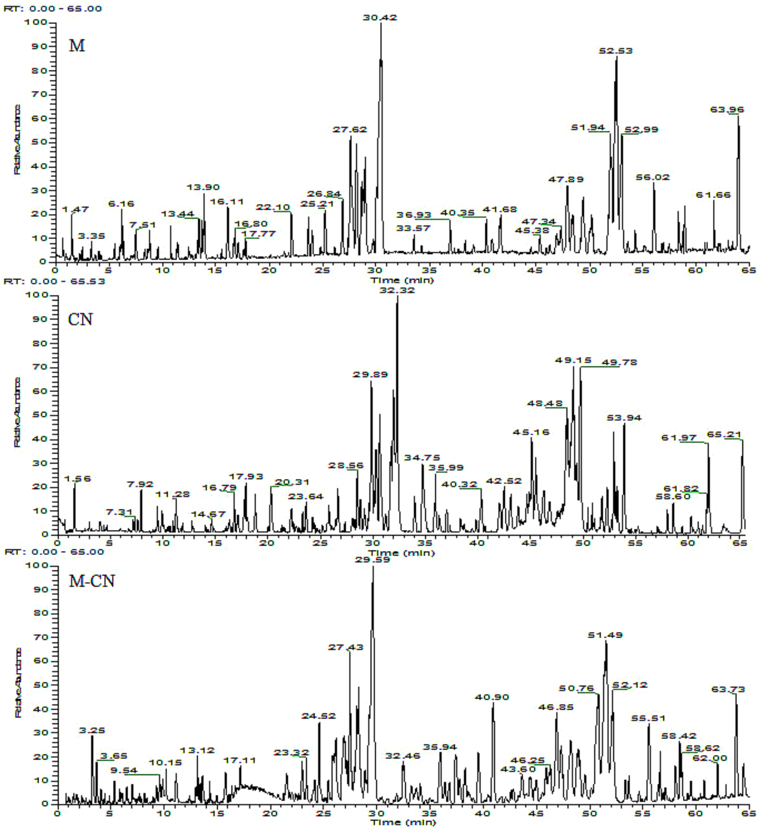

Figure S2. BPC chromatograms of YS separated on T3 column in different solvent systems. M: methanol; CN: CH_3CN.

Column: Waters ACQUITY UPLC® T_3 (2.1 × 100 mm, 1.8 μm). Mobile phase: H_2O (A) and FA-ACN (0.1:50, v/v) (B); H_2O (A) and FA-MeOH (0.1:50, v/v) (B); H_2O (A) and FA-MeOH-ACN (0.1:50:50, v/v/v) (B). Gradient program: 0-15 min, 12-24% B; 15-30 min, 24-32% B; 30-42 min, 32-40% B; 42-47 min, 40-60% B; 47-65 min, 60-95% B. Flow rate: 0.3 mL/min. Column temperature: 30°C. Injection volume: 3 μL. ESI-Q-Orbitrap MS mode: positive ion mode.

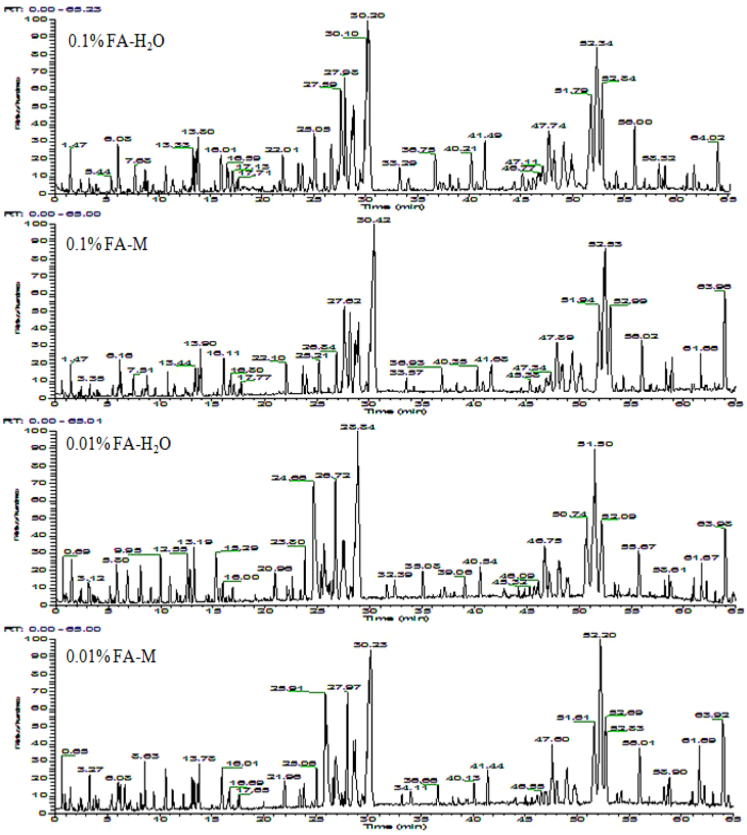

Figure S3. BPC chromatograms of YS separated on T3 column with different concentrations of formic acid (FA).

Column: Waters ACQUITY UPLC® T3 (2.1 × 100 mm, 1.8 μm). Mobile phase: FA-H_2O (0.1:100, v/v) (A) and MeOH-ACN (50:50, v/v) (B); H_2O (A) and FA-MeOH-ACN (0.1:50:50, v/v/v) (B); FA-H_2O (0.01:100, v/v) (A) and MeOH-ACN (50:50, v/v) (B); H_2O (A) and FA-MeOH-ACN (0.01:50:50, v/v/v) (B). Gradient program: 0-15 min, 12-24% B; 15-30 min, 24-32% B; 30-42 min, 32-40% B; 42-47 min, 40-60% B; 47-65 min, 60-95% B. Flow rate: 0.3 mL/min. Column temperature: 30°C. Injection volume: 3 μL. ESI-Q-Orbitrap MS mode: positive ion mode.

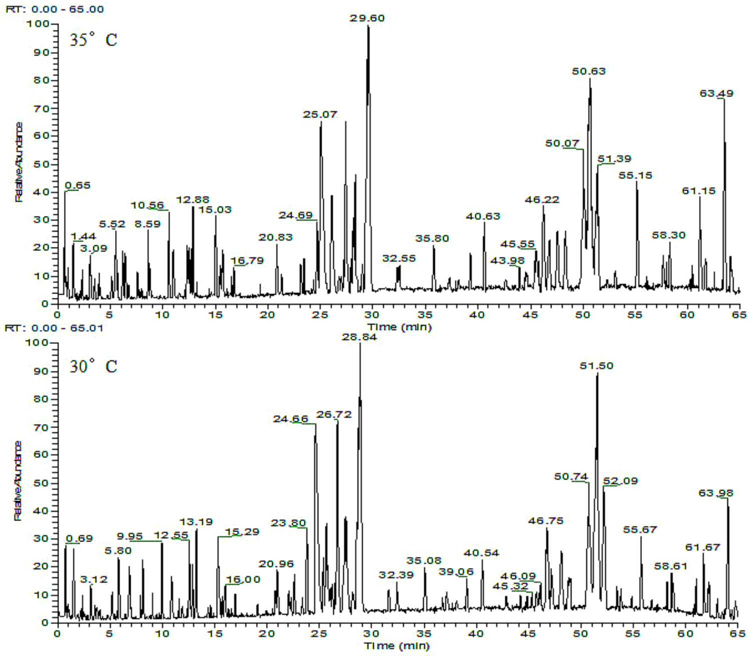

Figure S4. BPC chromatograms of YS separated on T3 column at different temperature (30°C and 35°C). Column: Waters ACQUITY UPLC® T_3 (2.1 × 100 mm, 1.8 μm). Mobile phase: H_2O (A) and MeOH-ACN-HCOOH (1:50:50, v/v/v) (B). Gradient program: 0-15 min, 12-24% B; 15-30 min, 24-32% B; 30-42 min, 32-40% B; 42-47 min, 40-60% B; 47-65 min, 60-95% B. Flow rate: 0.3 mL/min. Column temperature: 30°C and 35°C. Injection volume: 3 μL. ESI-Q-Orbitrap MS mode: positive ion mode.

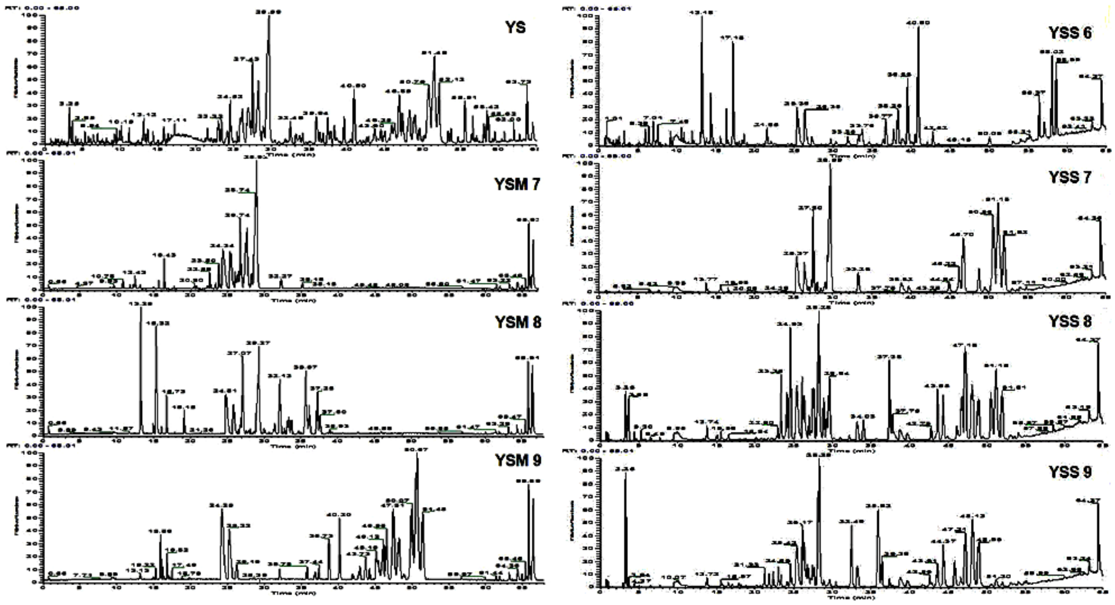

Figure S5. BPC for YS, YSESs 6–9 and YSEMs 7–9 by OC-MS in positive ion mode.

Column: Waters ACQUITY UPLC® T_3 (1.8 μm, 2.1 × 100 mm). Mobile phase: H_2O (A) and FA-MeOH-ACN (0.1:50:50, v/v/v) (B). Gradient program: 0-15 min, 12-24% B; 15-30 min, 24-32% B; 30-42 min, 32-40% B; 42-47 min, 40-60% B; 47-65 min, 60-95% B. Flow rate: 0.3 mL/min. Column temperature: 30°C. Injection volume: 3 μL. ESI-Q-Orbitrap MS mode: positive ion mode.

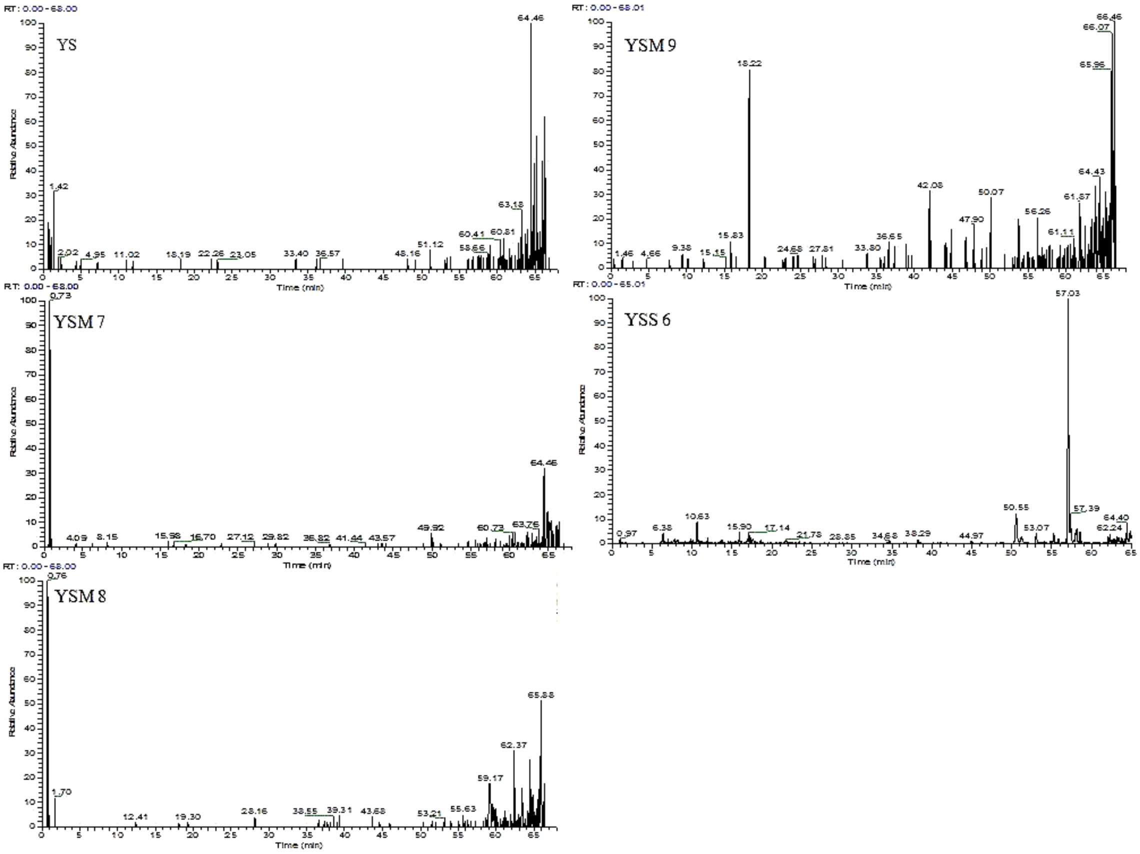

Figure S6. EIC of *m*/*z* 741.4420 (t_R: 57.03 min) from YS, YSM 7–9 and YSS 6

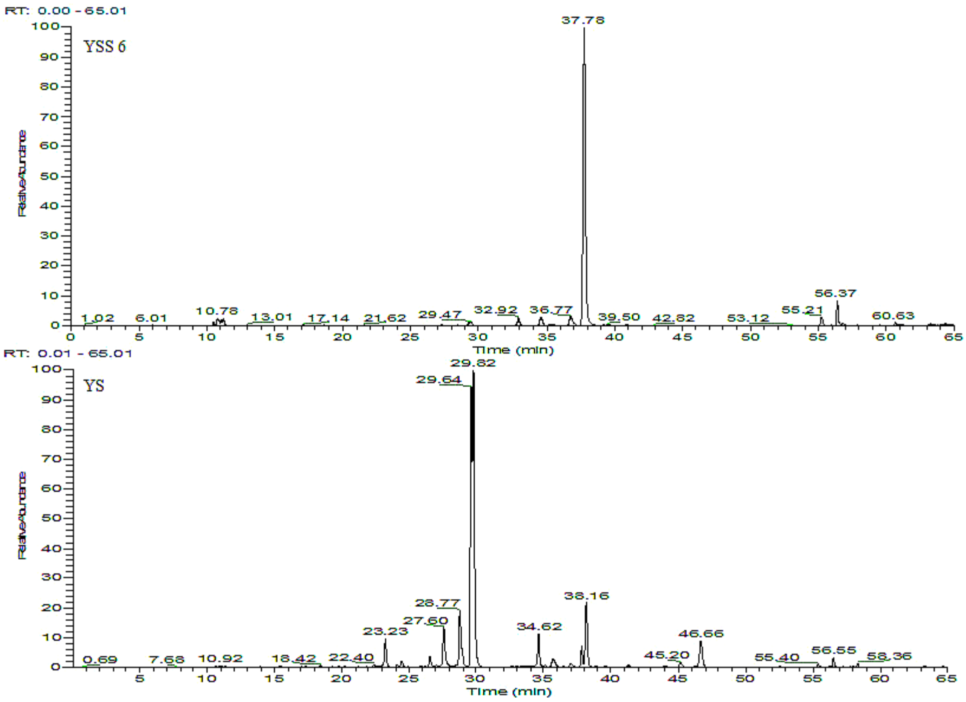

Figure S7. EIC of *m*/*z* 755.4223 (t_R: 37.76 min) from YSS 6 and YS

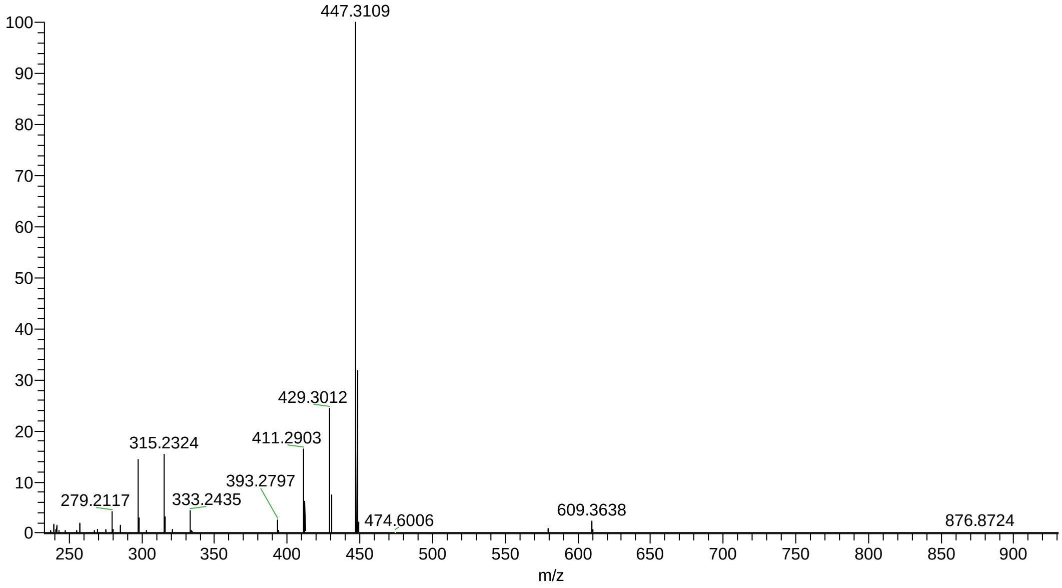

Figure S8. The MS/MS spectrum of peak **10**

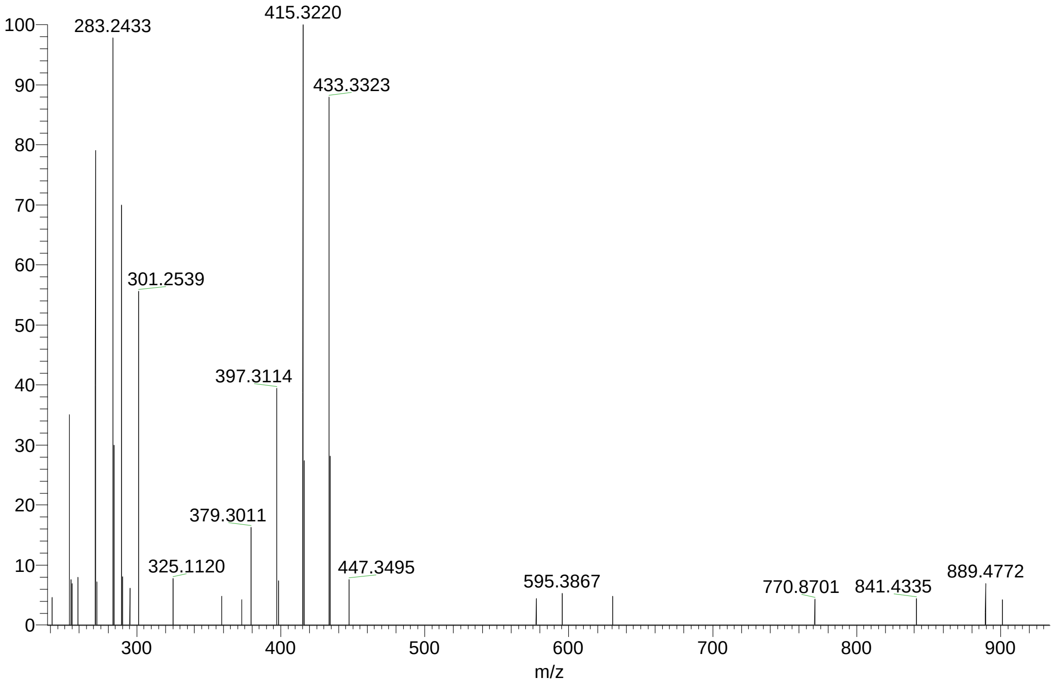

Figure S9. The MS/MS spectrum of peak **14**

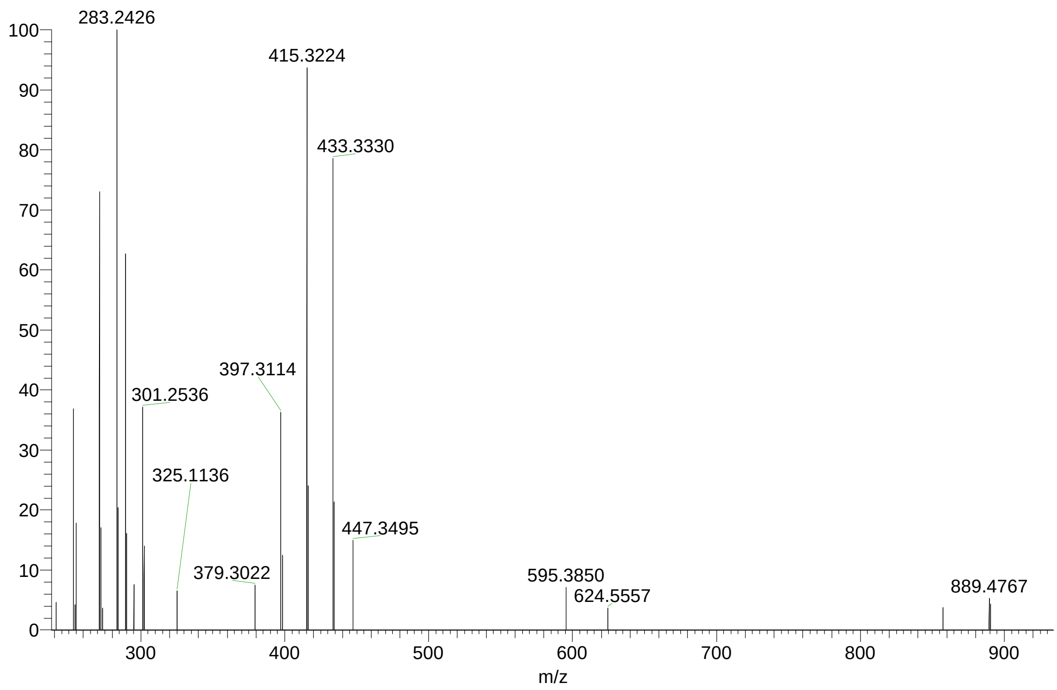

Figure S10. The MS/MS spectrum of peak **15**

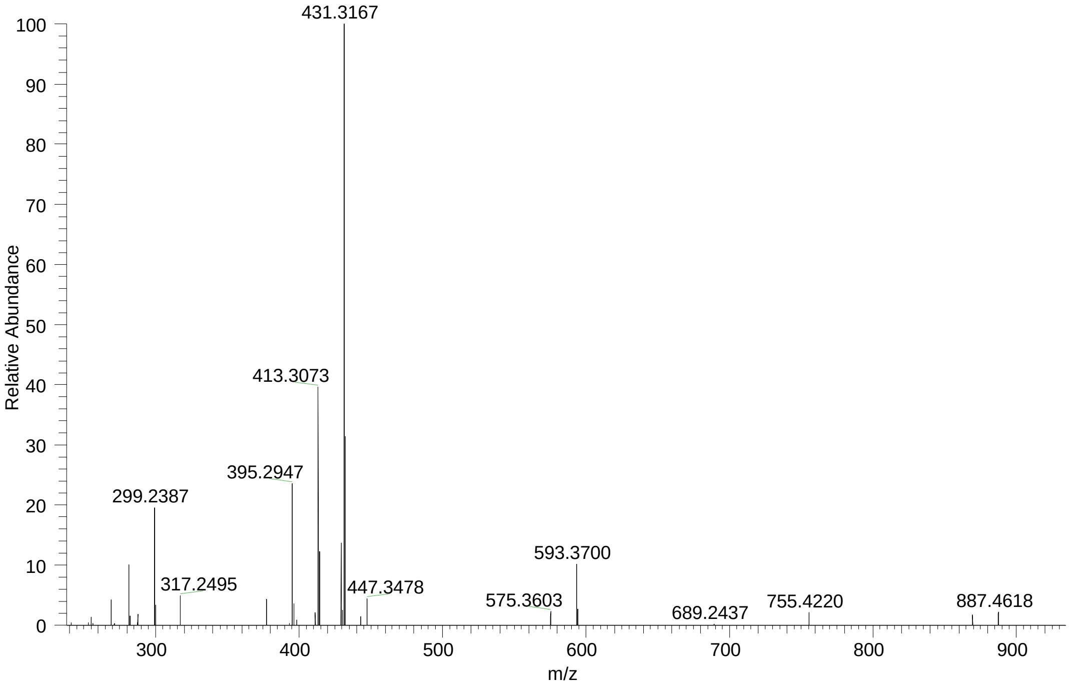

Figure S11. The MS/MS spectrum of peak **27**

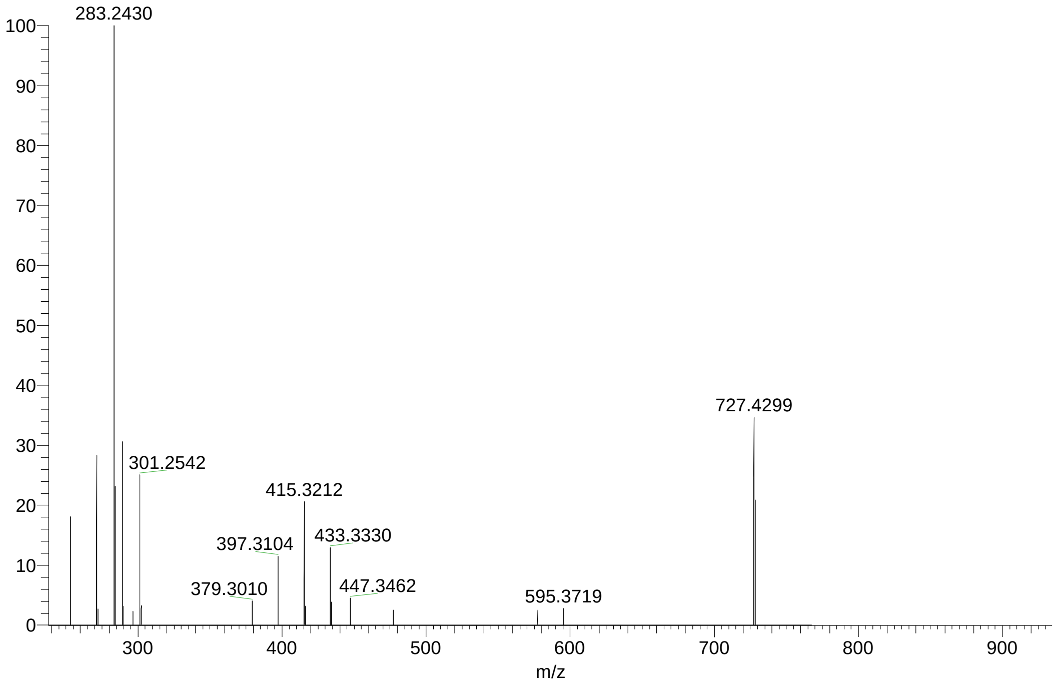

Figure S12. The MS/MS spectrum of peak **34**

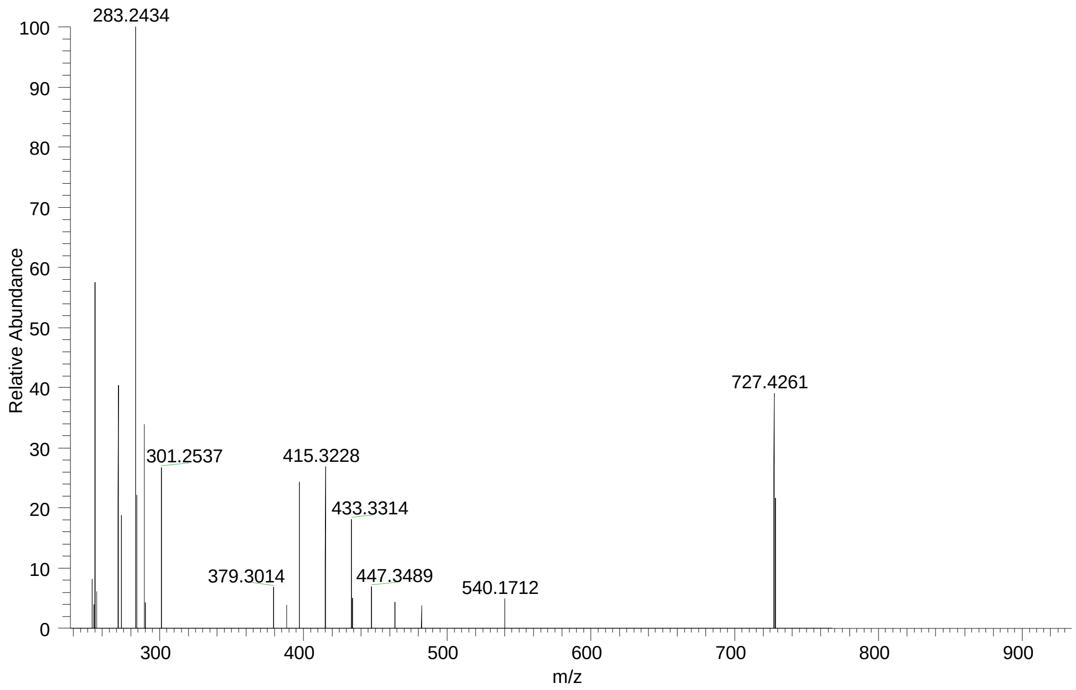

Figure S13. The MS/MS spectrum of peak **36**

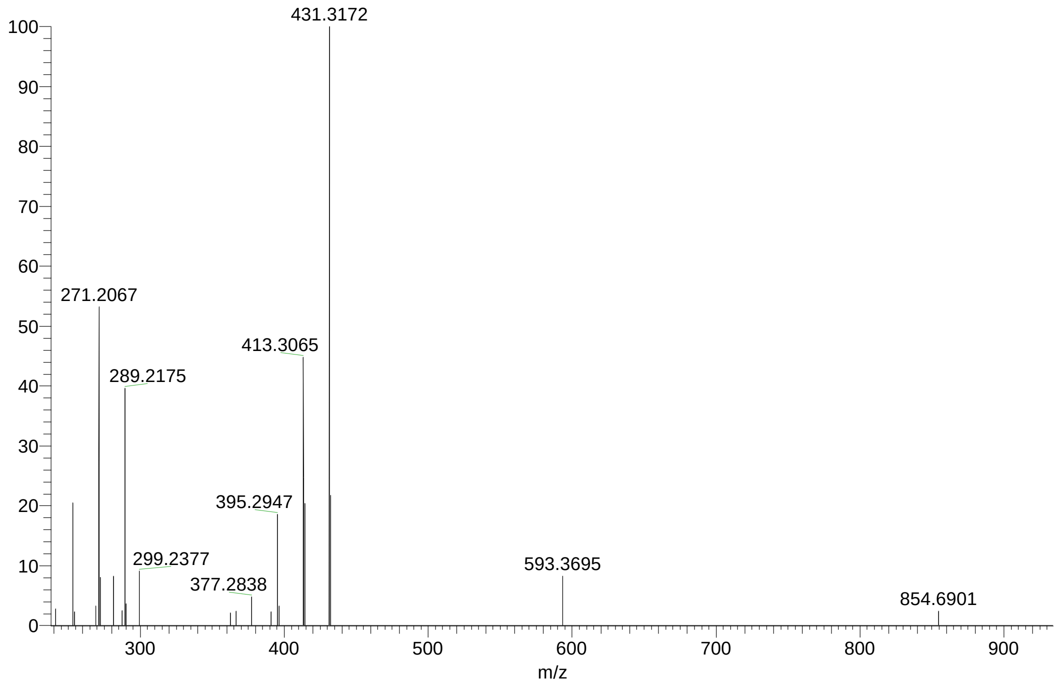

Figure S14. The MS/MS spectrum of peak **38**

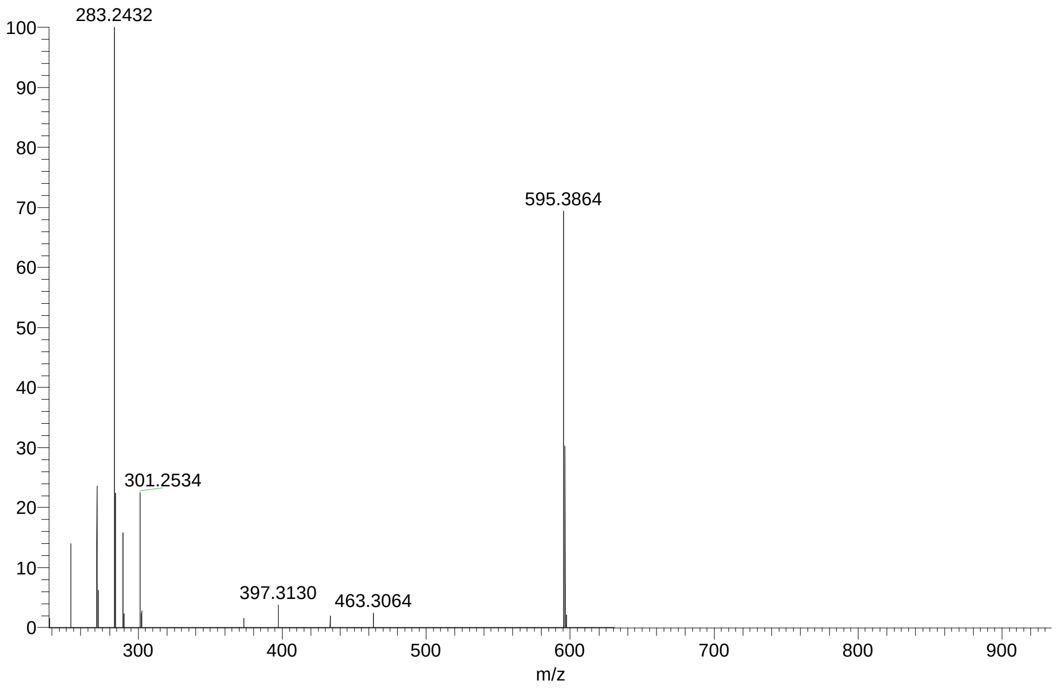

Figure S15. The MS/MS spectrum of peak **40**

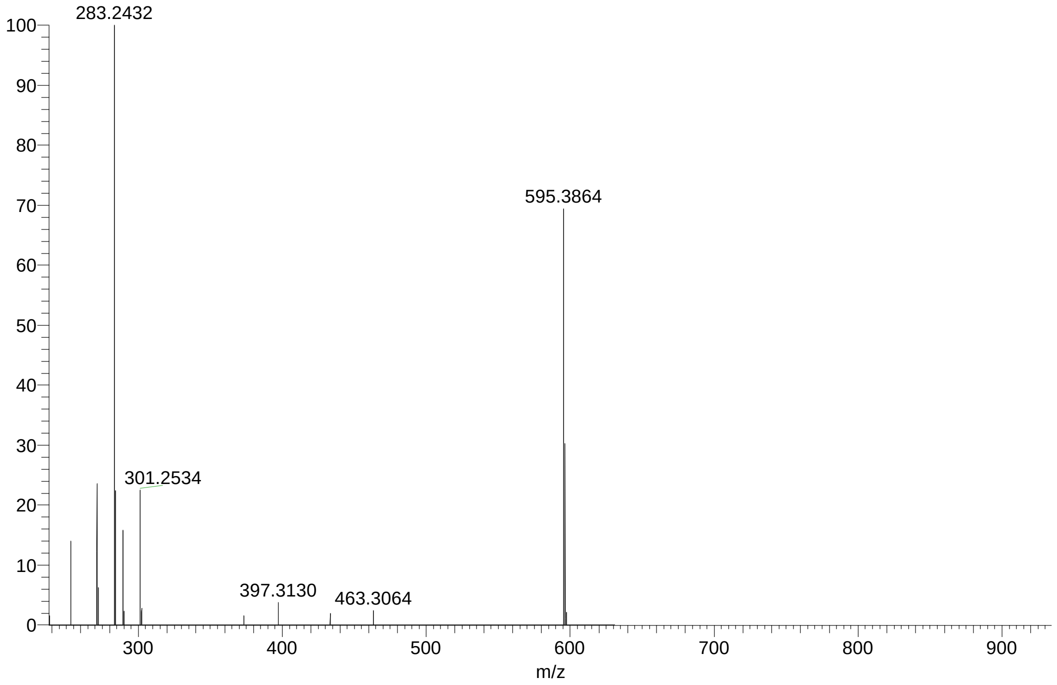

Figure S16. The MS/MS spectrum of peak **41**

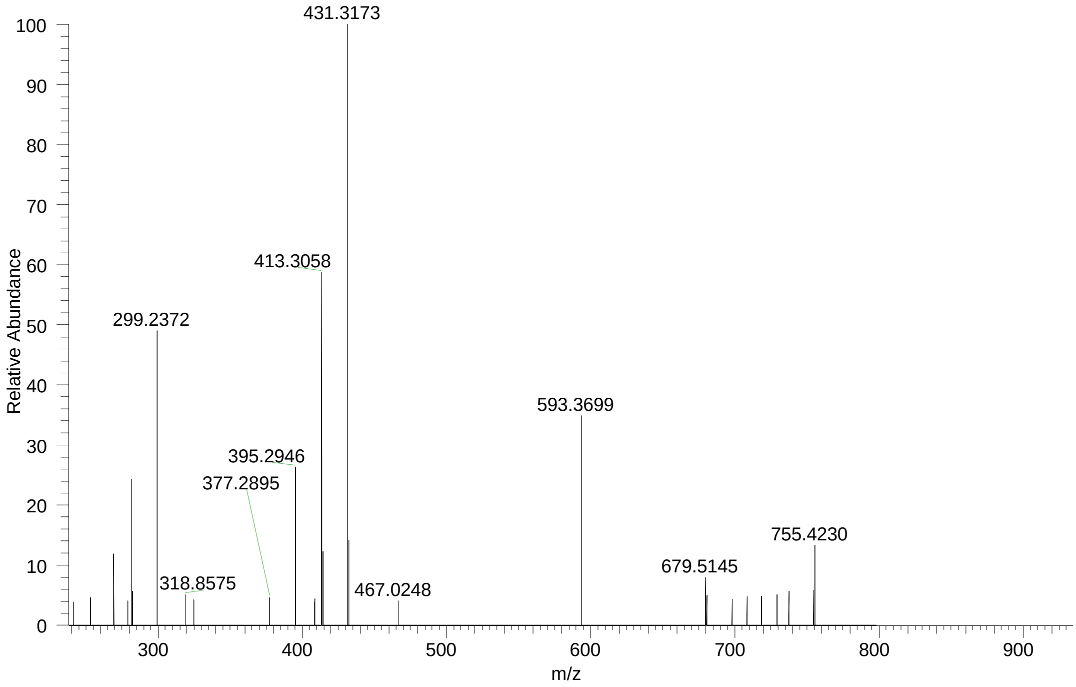

Figure S17. The MS/MS spectrum of peak **51**

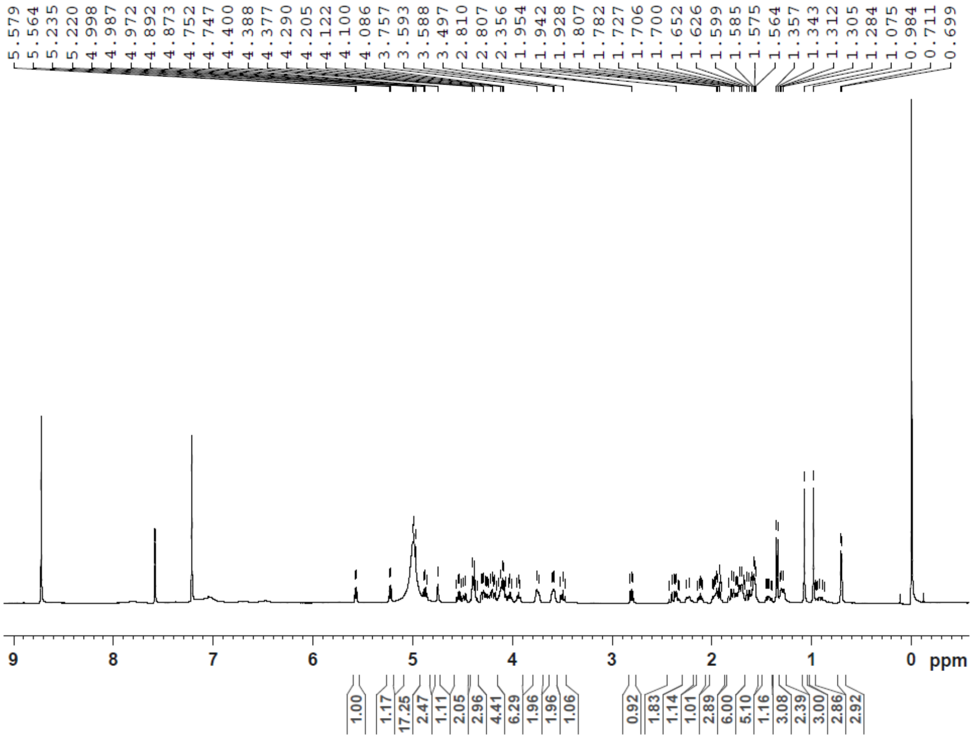

Figure S18. ^{1}H NMR (500MHz, C_5D_5N) spectrum of compound **10**

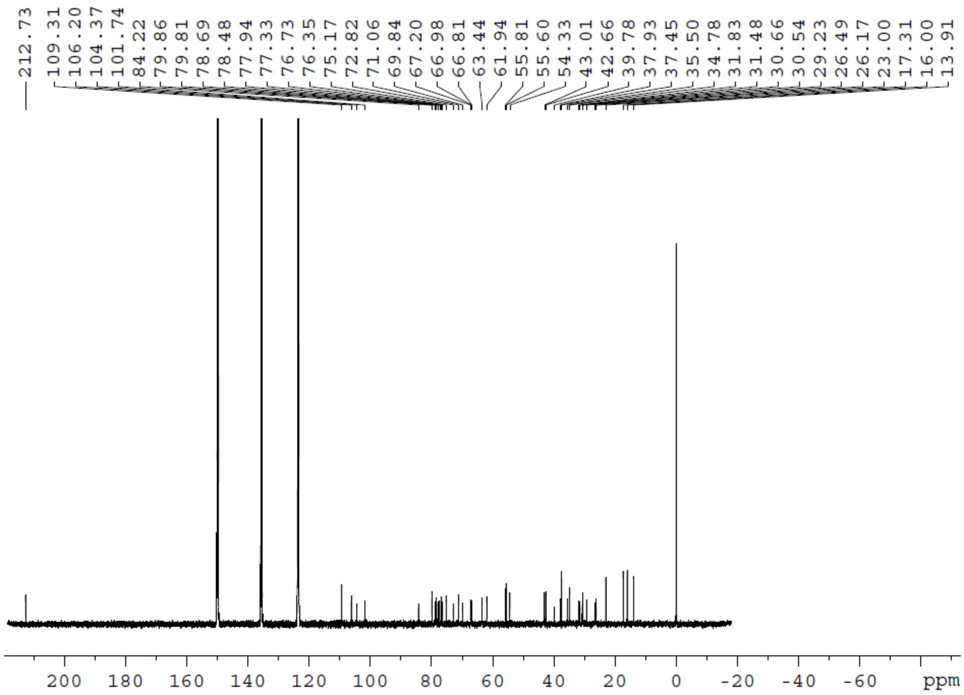

Figure S19. ^{13}C NMR (125MHz, C_5D_5N) spectrum of compound **10**

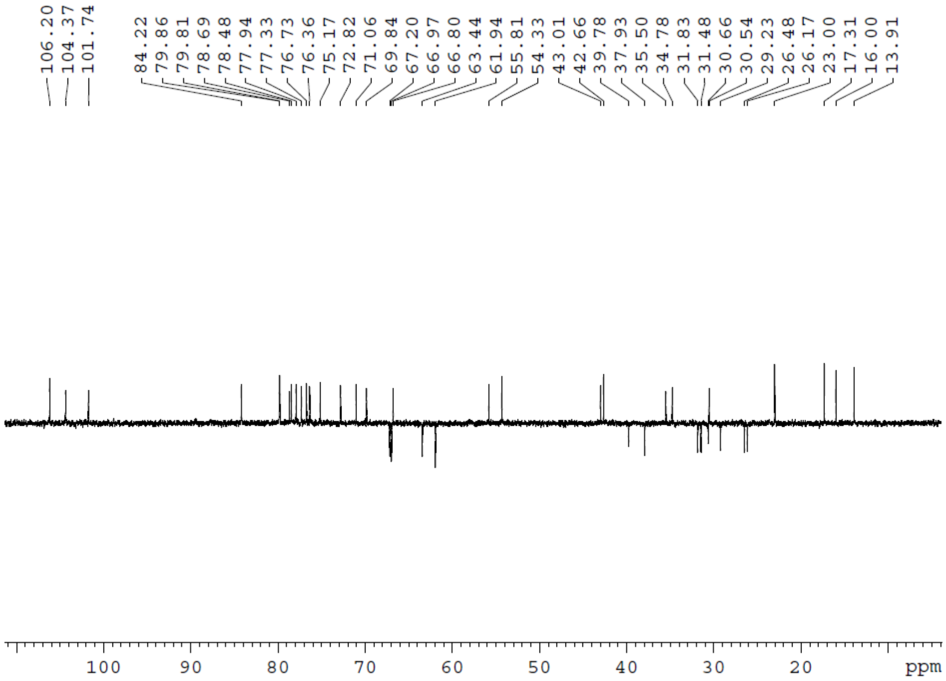

Figure S20. The DEPT 135 (C_5D_5N) spectrum of compound **10**

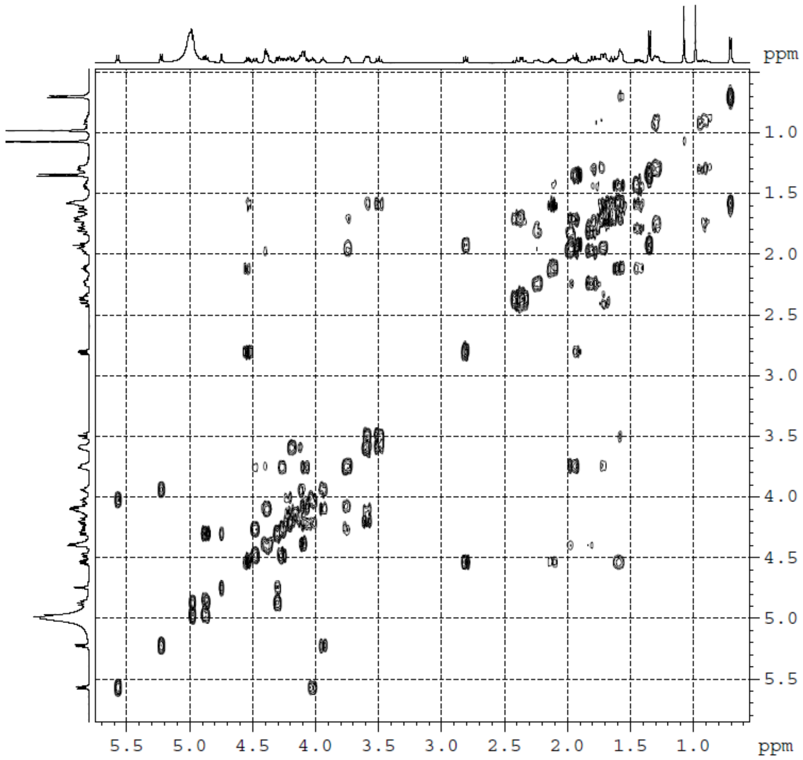

Figure S21. The ^{1}H ^{1}H COSY (C_5D_5N) spectrum of compound **10**

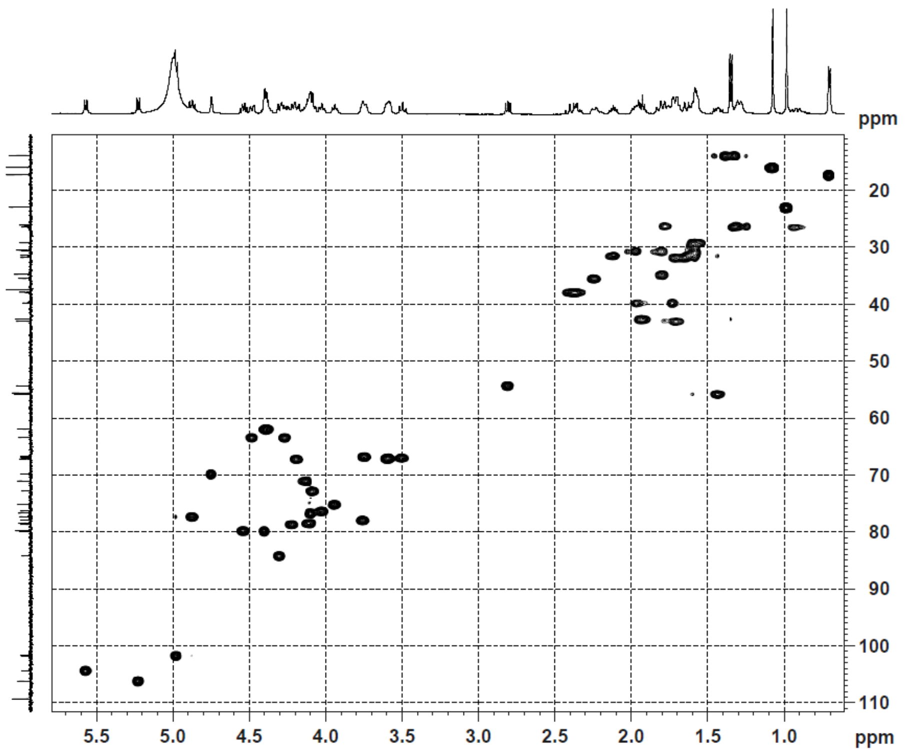

Figure S22. The HSQC (C_5D_5N) spectrum of compound **10**

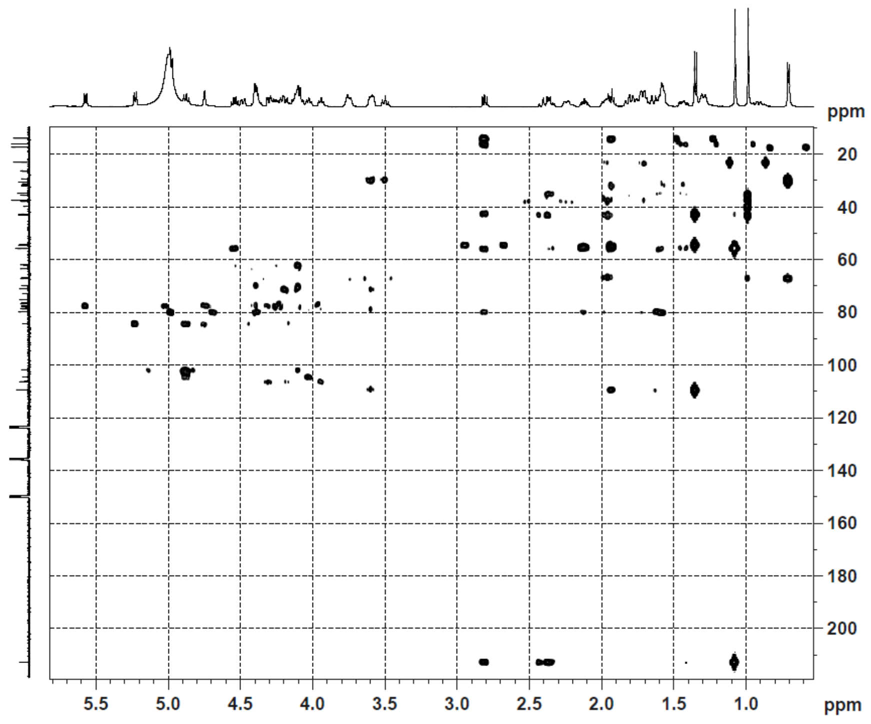

Figure S23. The HMBC (C_5D_5N) spectrum of compound **10**

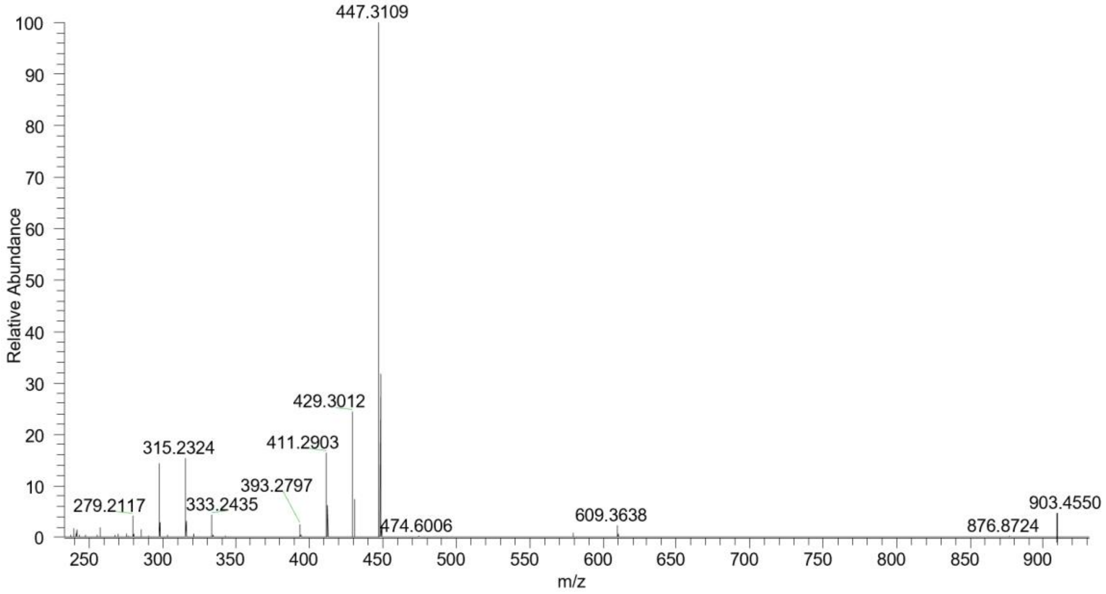

Figure S24. The ESI-Q-Orbitrap MS spectrum of compound **10**

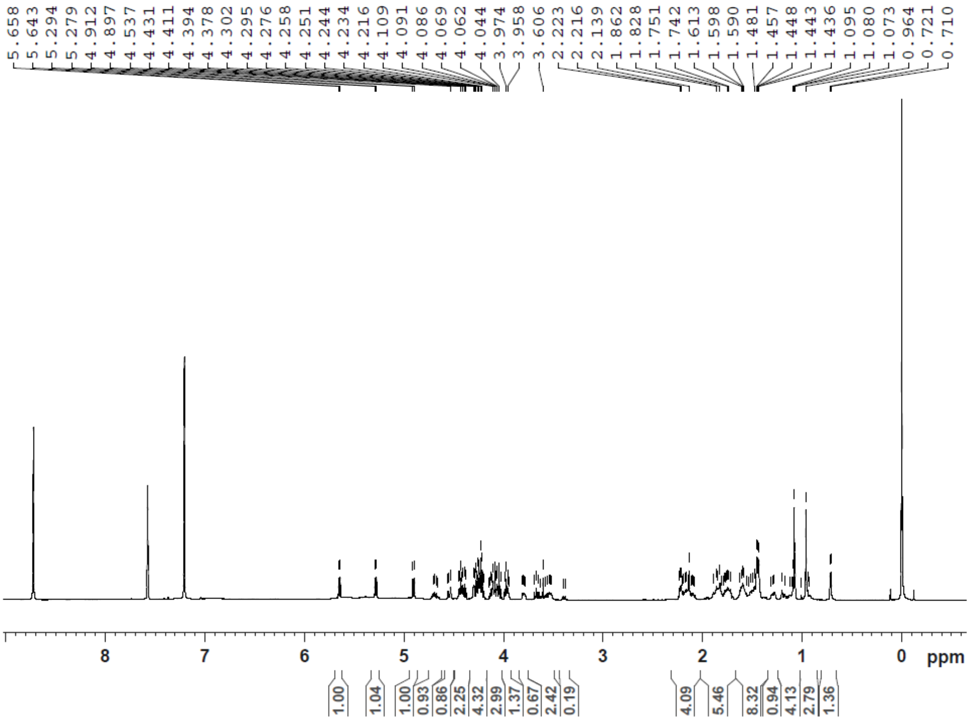

Figure S25. ^{1}H NMR (500MHz, C_5D_5N) spectrum of compound **14/15**

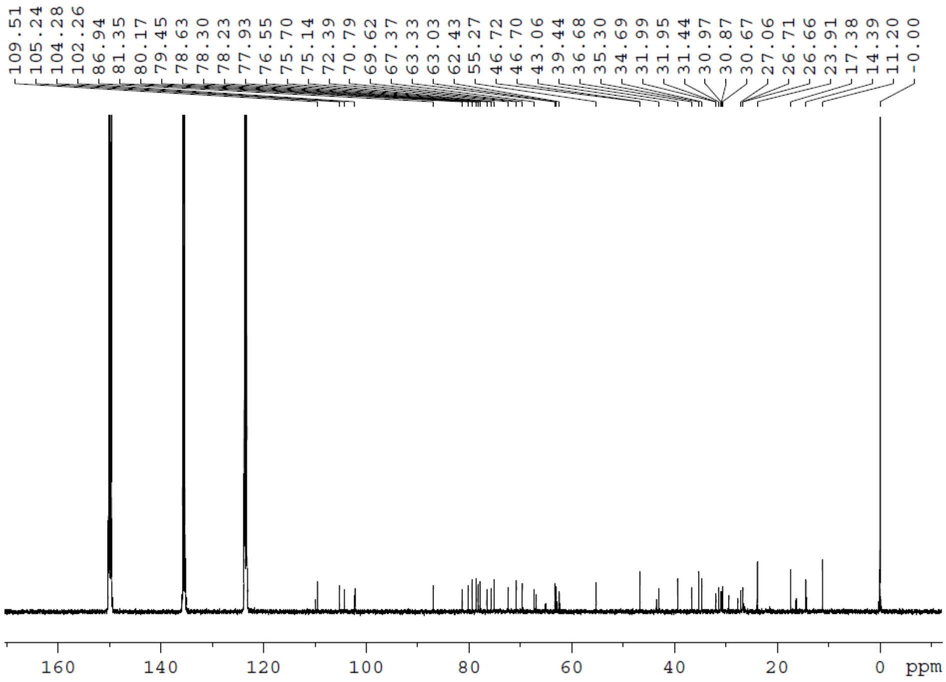

Figure S26. ^{13}C NMR (125MHz, C_5D_5N) spectrum of compound **14/15**

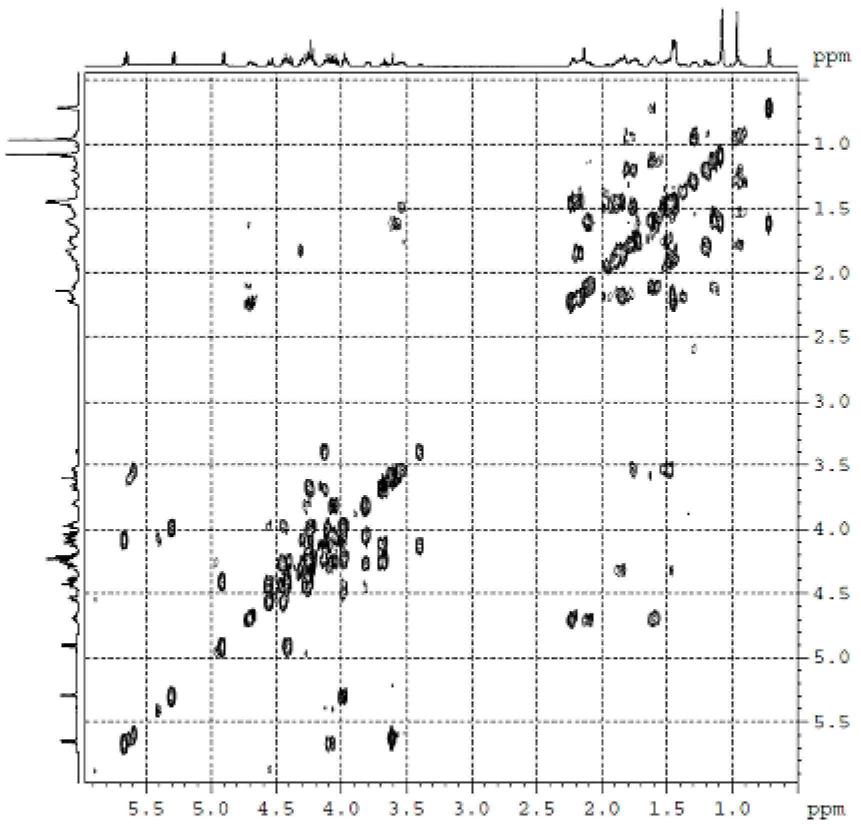

Figure S27. The ^{1}H ^{1}H COSY (C_5D_5N) spectrum of compound **14/15**

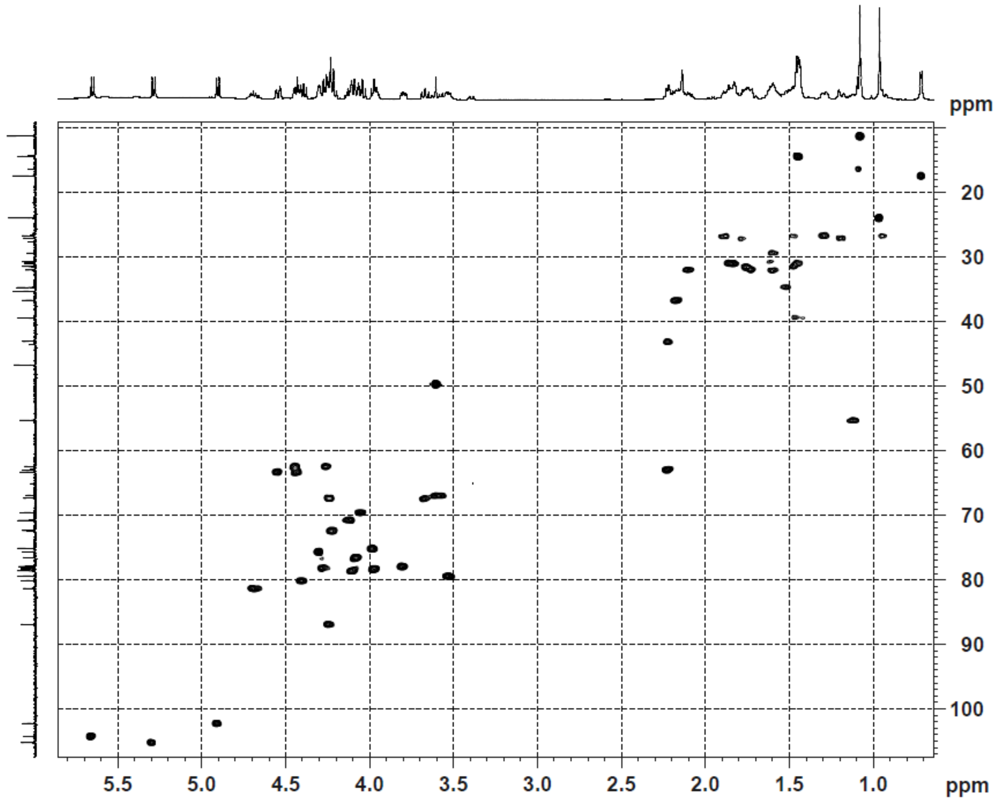

Figure S28. The HSQC (C_5D_5N) spectrum of compound **14/15**

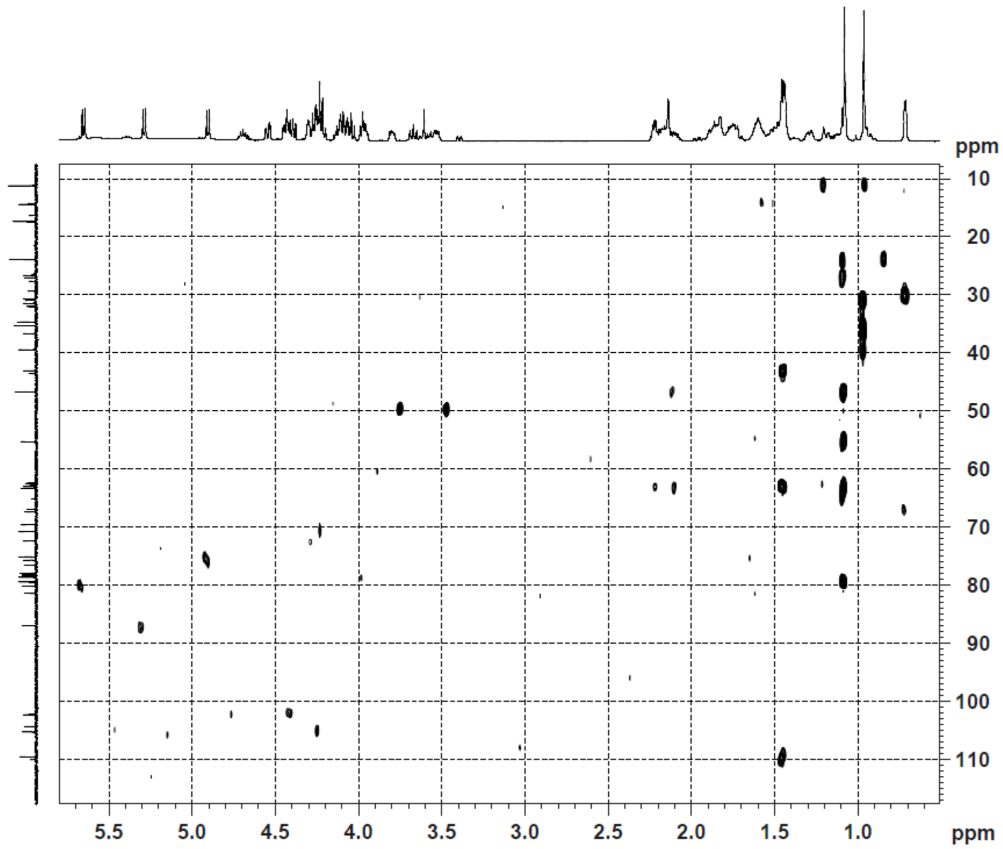

Figure S29. The HMBC (C_5D_5N) spectrum of compound **14/15**

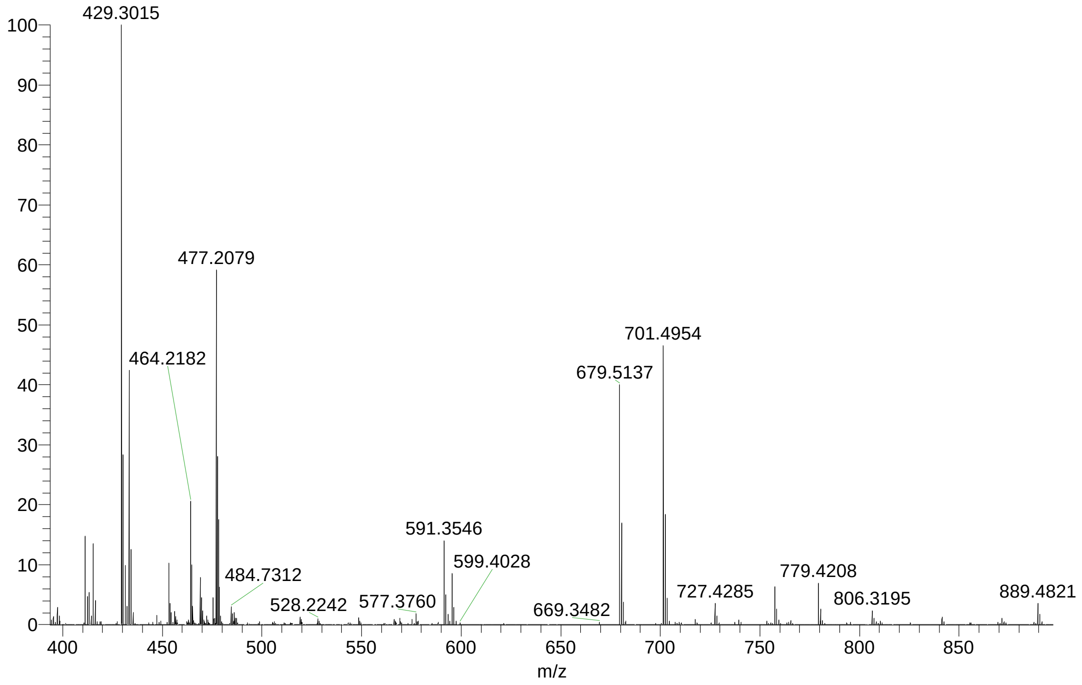

Figure S30. The ESI-Q-Orbitrap MS spectrum of compound **14/15**

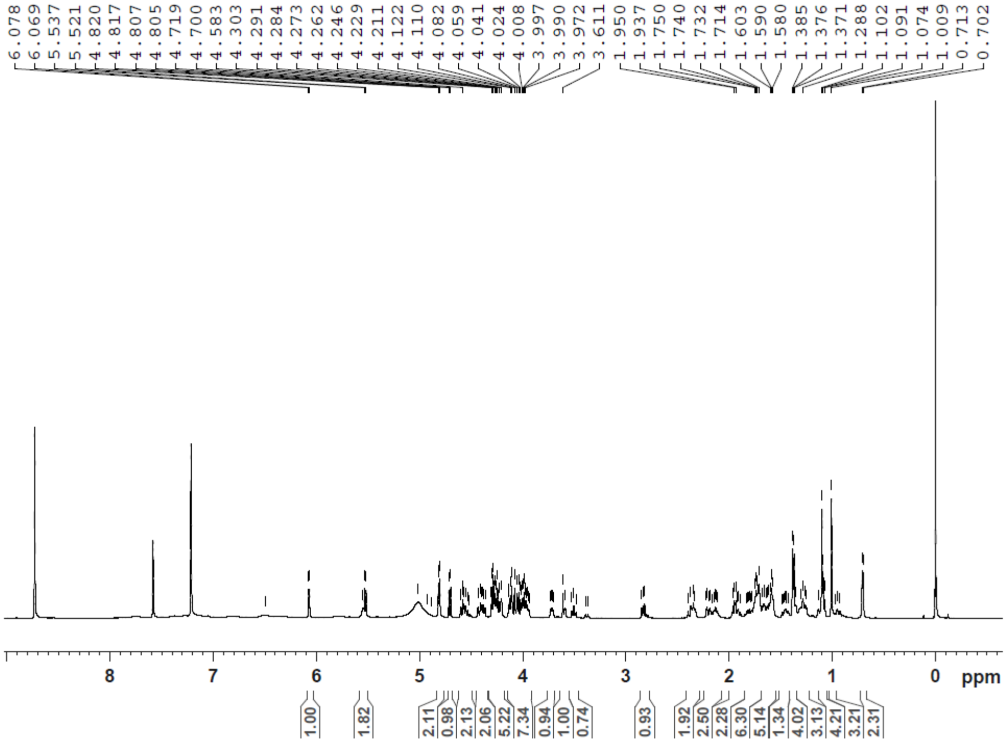

Figure S31. ^{1}H NMR (500MHz, C_5D_5N) spectrum of compound **27**

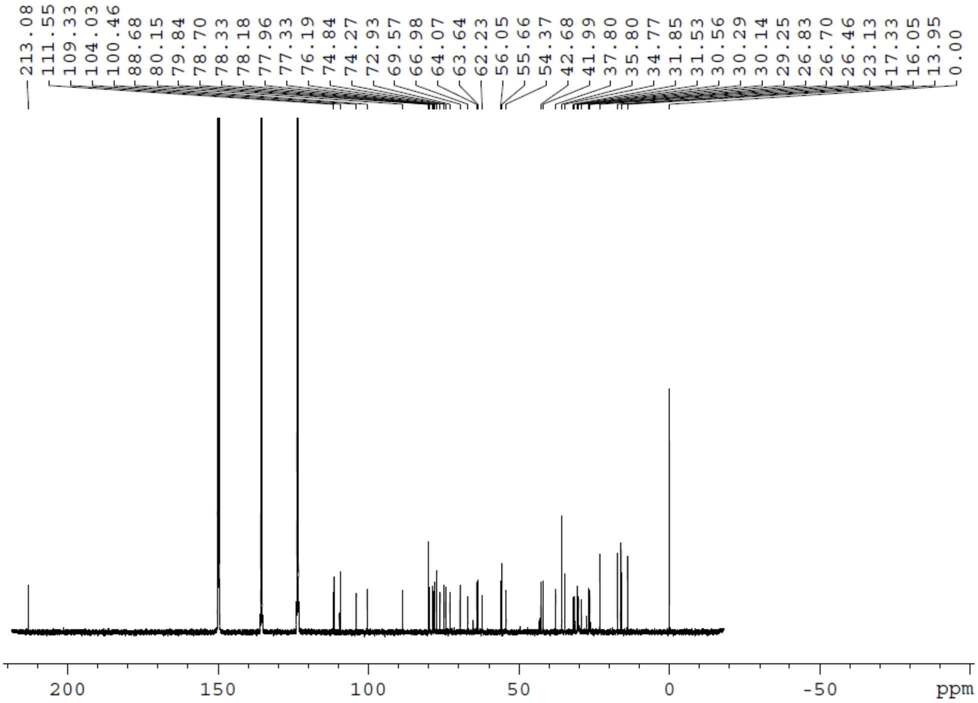

Figure S32. ^{13}C NMR (125MHz, C_5D_5N) spectrum of compound **27**

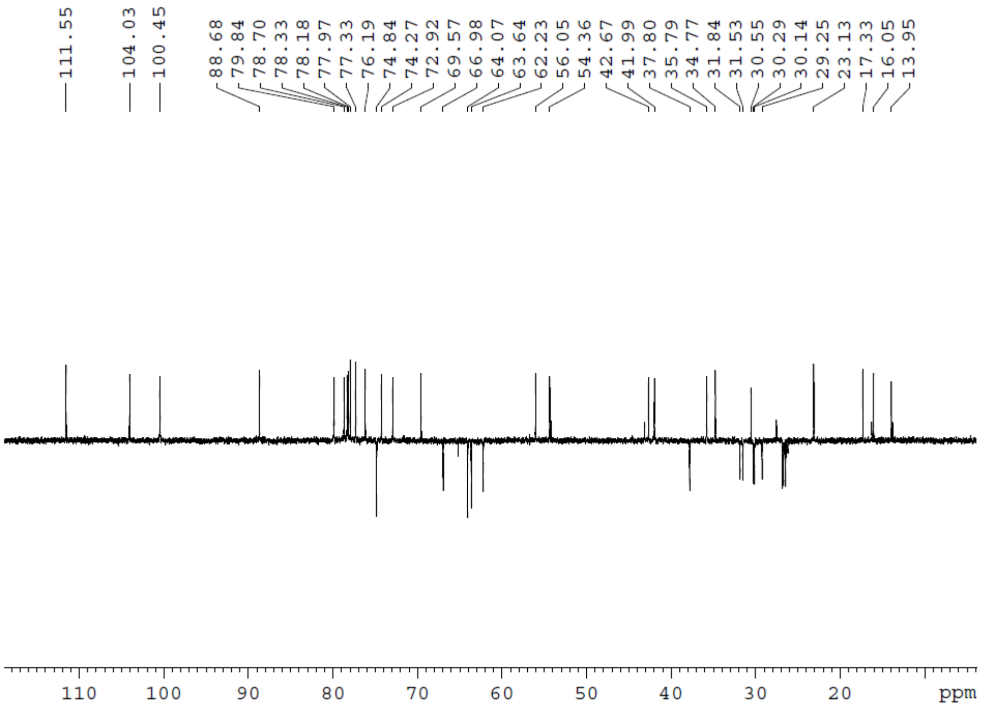

Figure S33. The DEPT 135 (C_5D_5N) spectrum of compound **27**

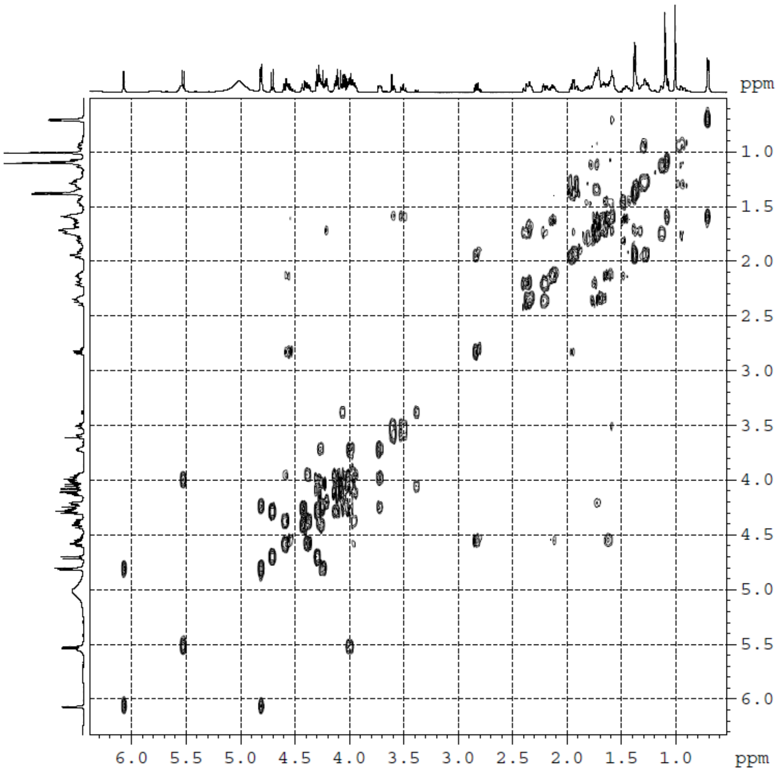

Figure S34. The ^1H ^1H COSY (C_5D_5N) spectrum of compound **27**

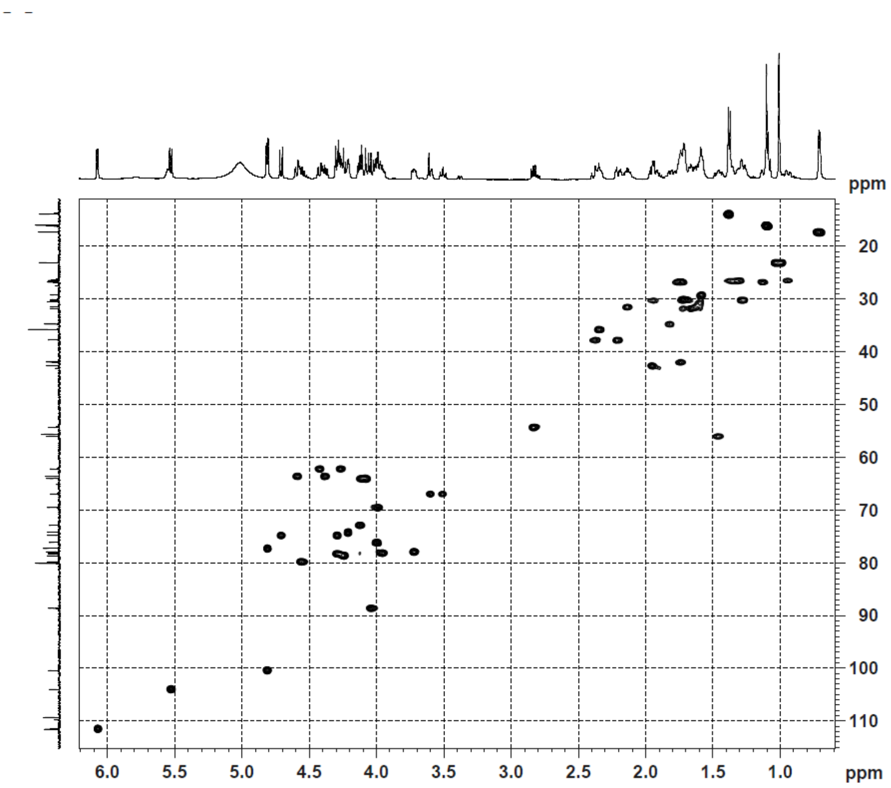

Figure S35. The HSQC (C_5D_5N) spectrum of compound **27**

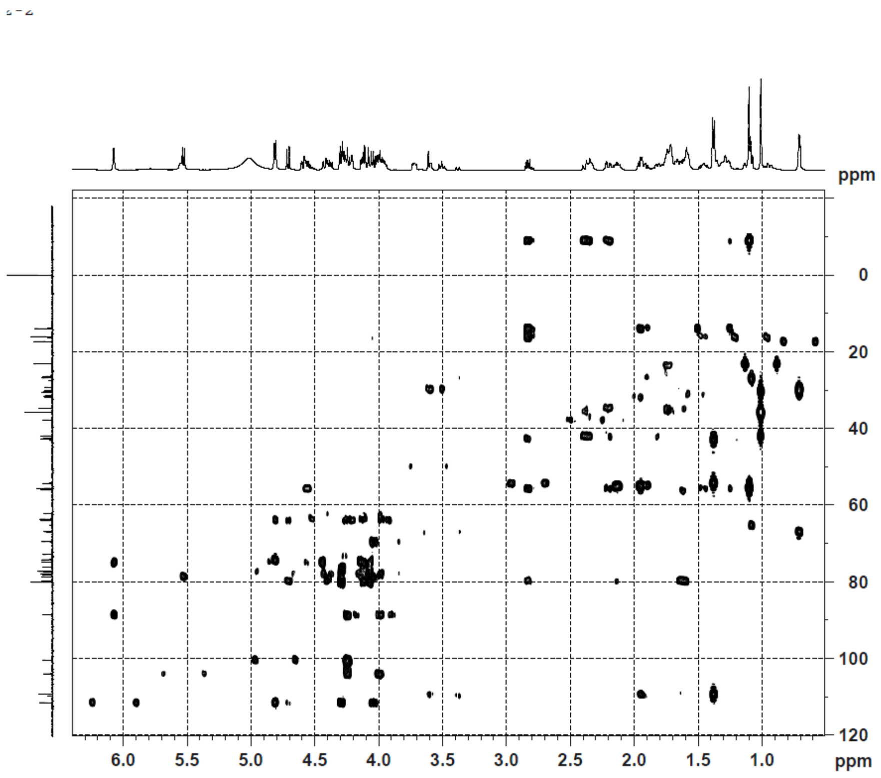

Figure S36. The HMBC (C_5D_5N) spectrum of compound **27**

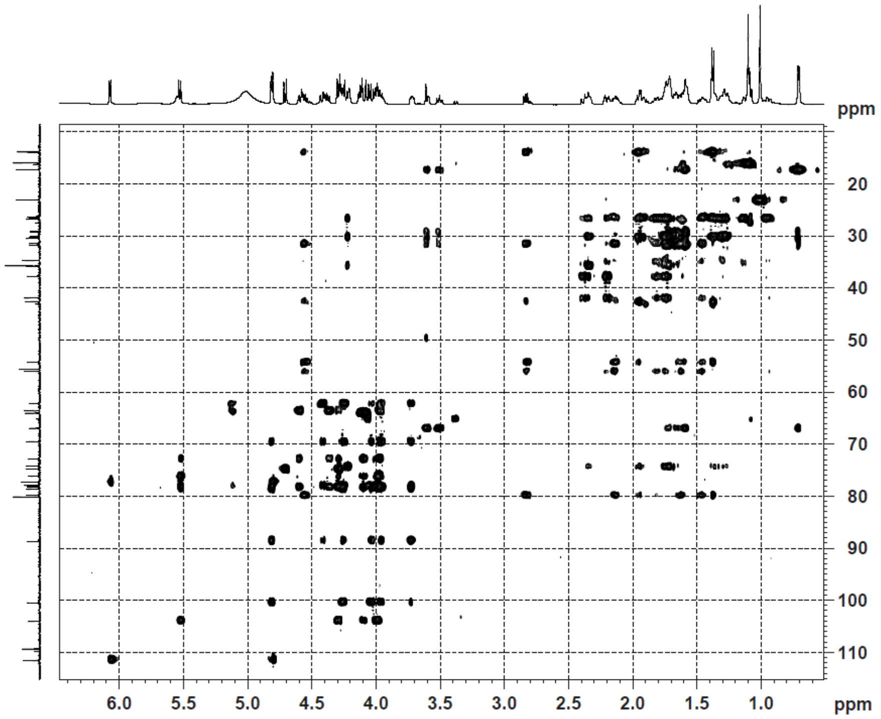

Figure S37. The HSQC-TOCSY (C_5D_5N) spectrum of compound **27**

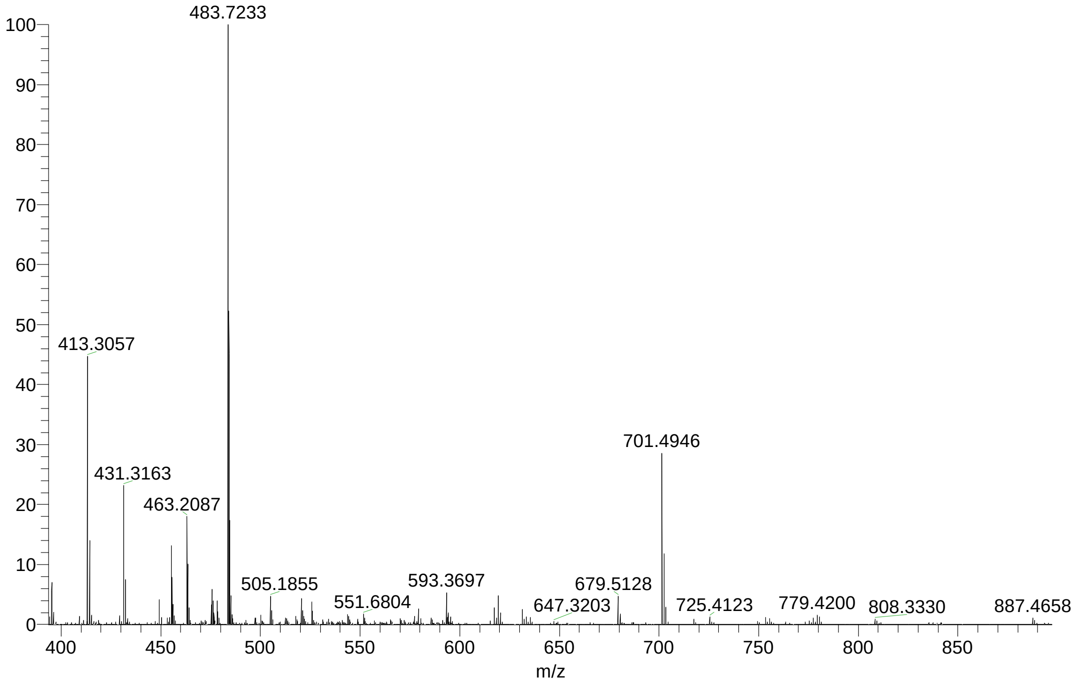

Figure S38. The ESI-Q-Orbitrap MS spectrum of compound **27**

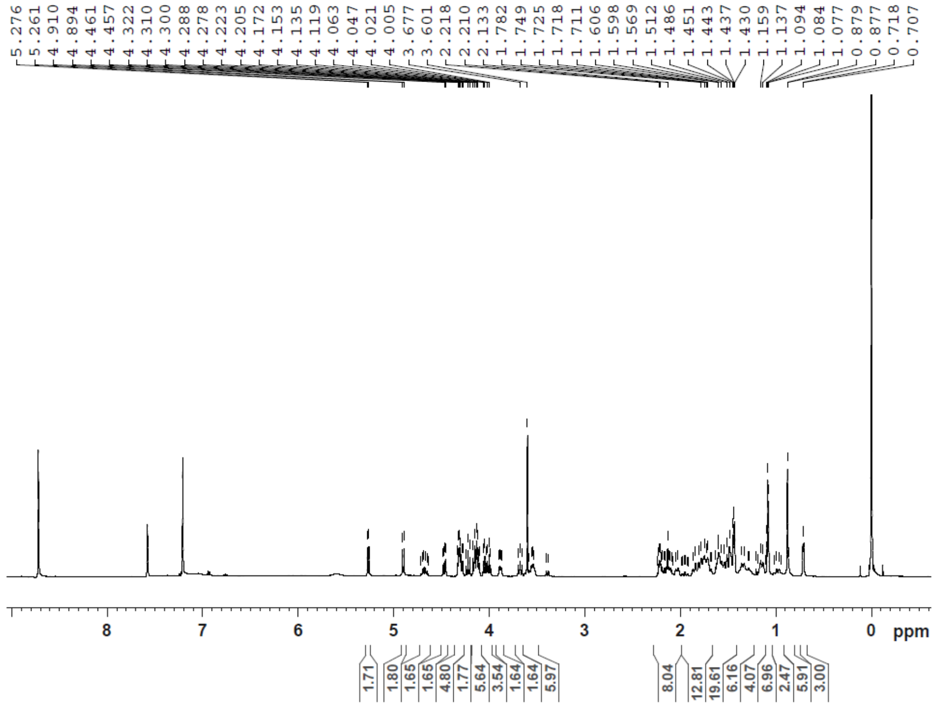

Figure S39. ^{1}H NMR (500MHz, C_5D_5N) spectrum of compound **34/36**

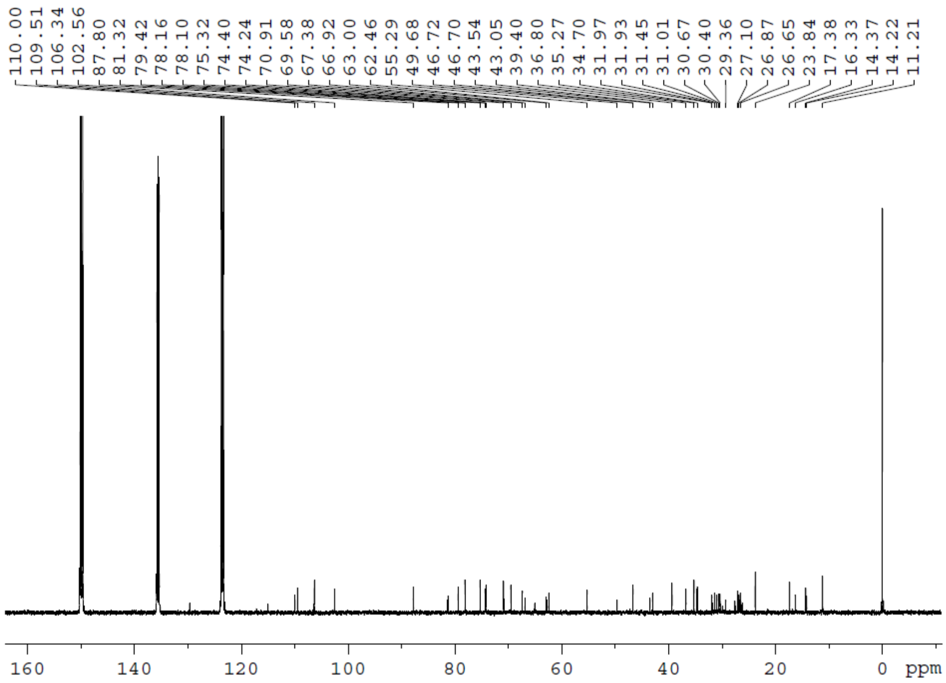

Figure S40. ^{13}C NMR (125MHz, C_5D_5N) spectrum of compound **34/36**

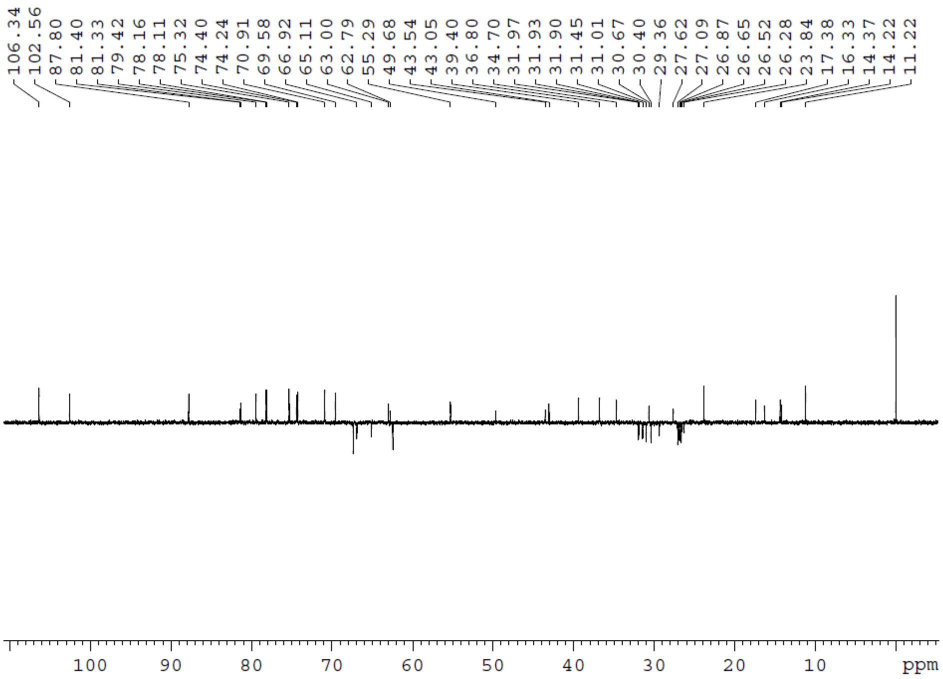

Figure S41. The DEPT 135 (C_5D_5N) spectrum of compound **34/36**

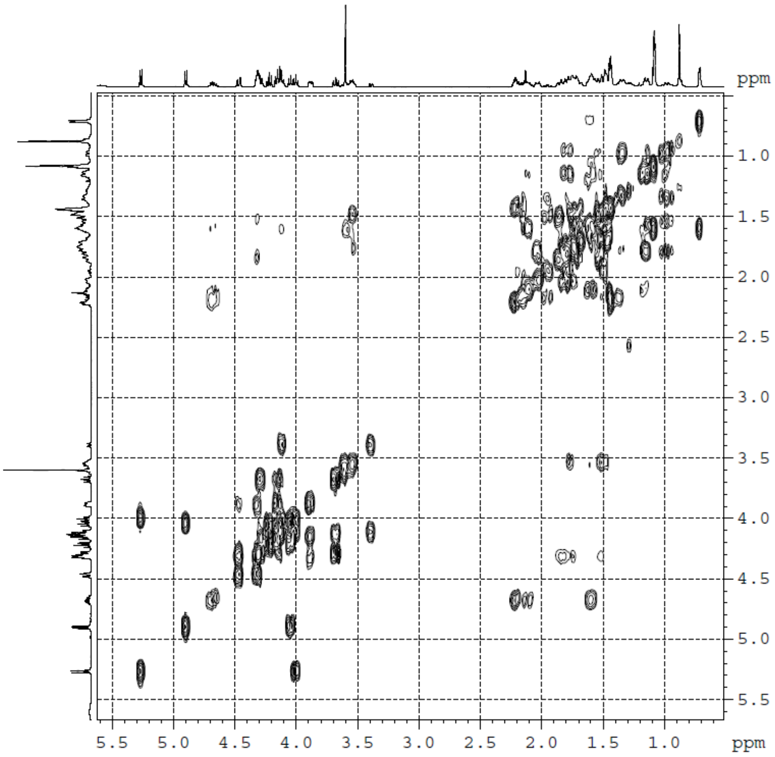

Figure S42. The ^{1}H ^{1}H COSY (C_5D_5N) spectrum of compound **34/36**

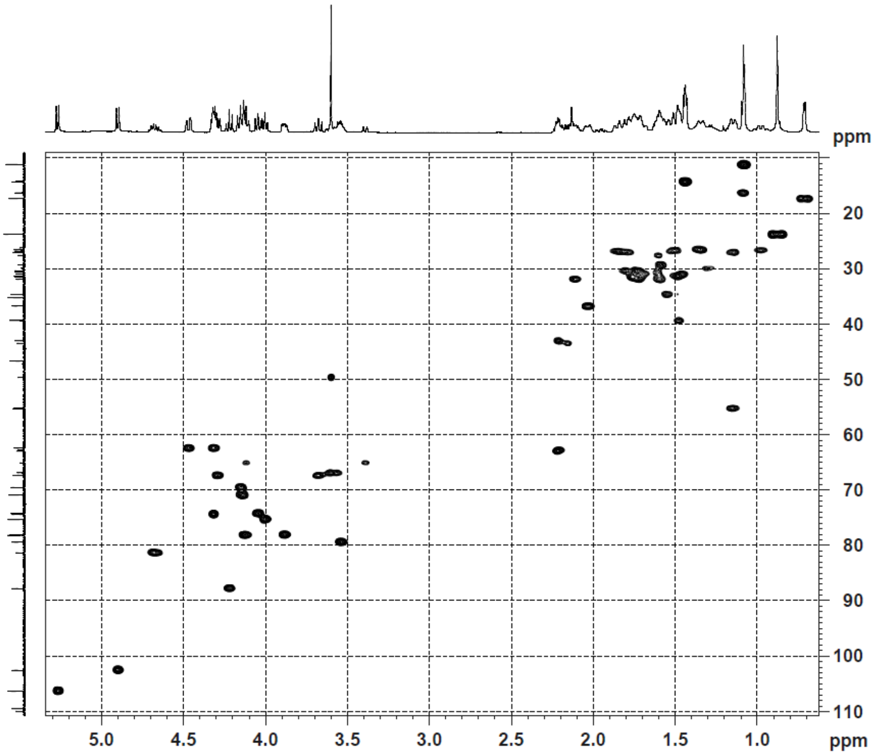

Figure S43. The HSQC (C_5D_5N) spectrum of compound **34/36**

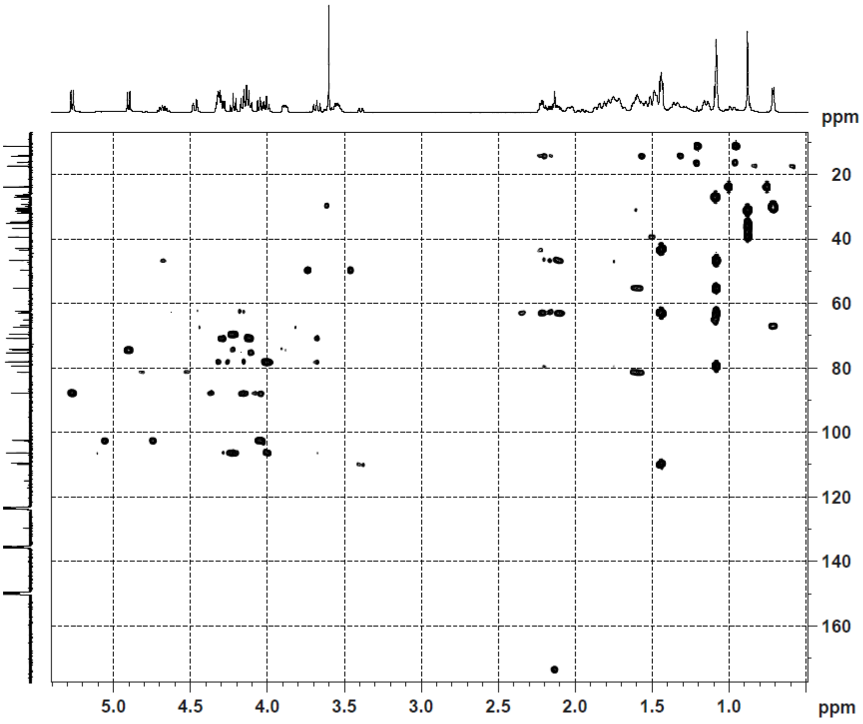

Figure S44. The HMBC (C_5D_5N) spectrum of compound **34/36**

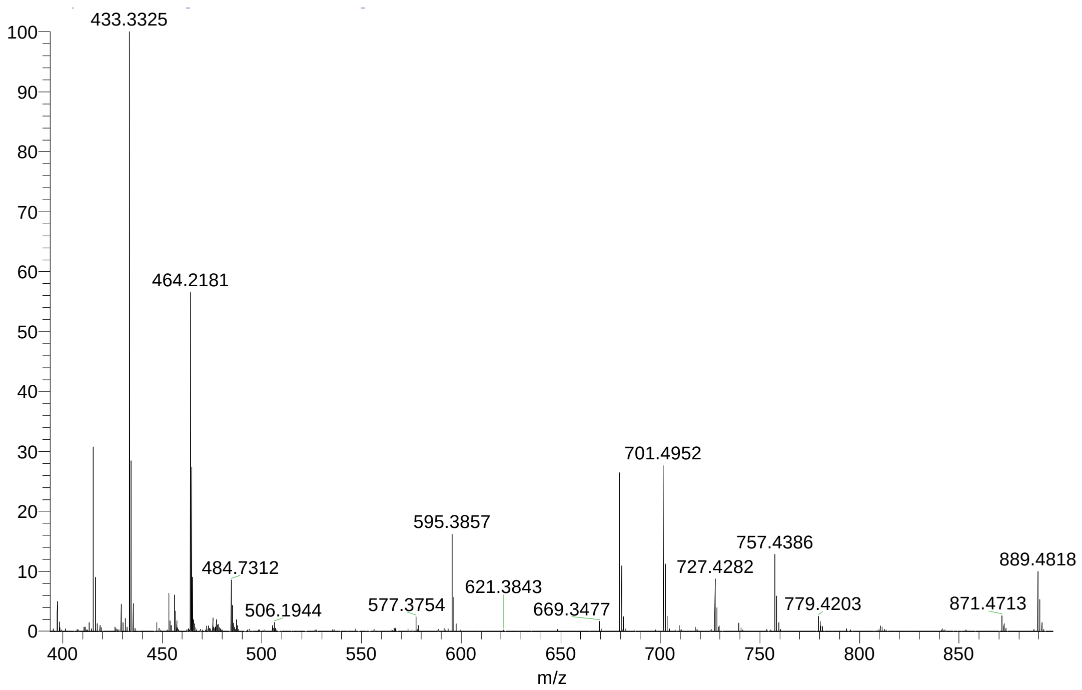

Figure S45. The ESI-Q-Orbitrap MS spectrum of compound **34/36**

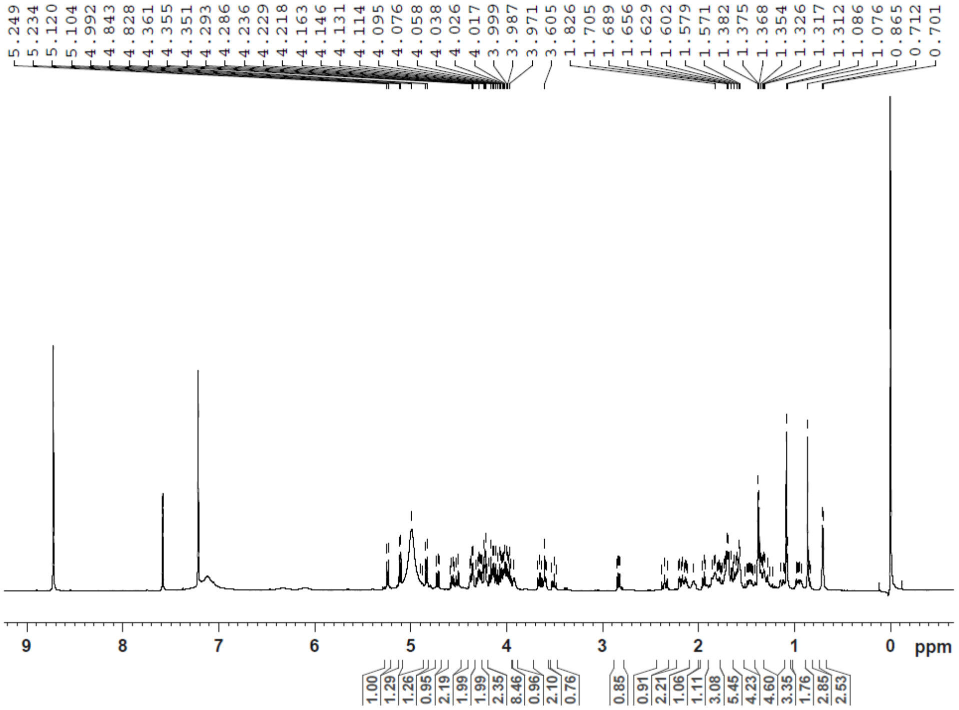

Figure S46. ^{1}H NMR (500MHz, C_5D_5N) spectrum of compound **38**

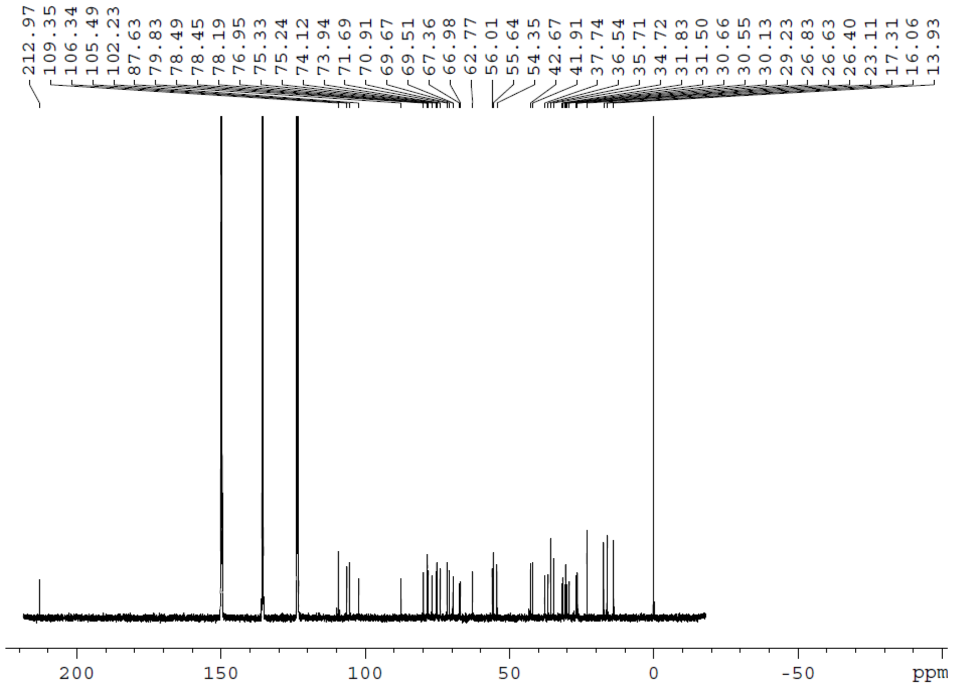

Figure S47. ^{13}C NMR (125MHz, C_5D_5N) spectrum of compound **38**

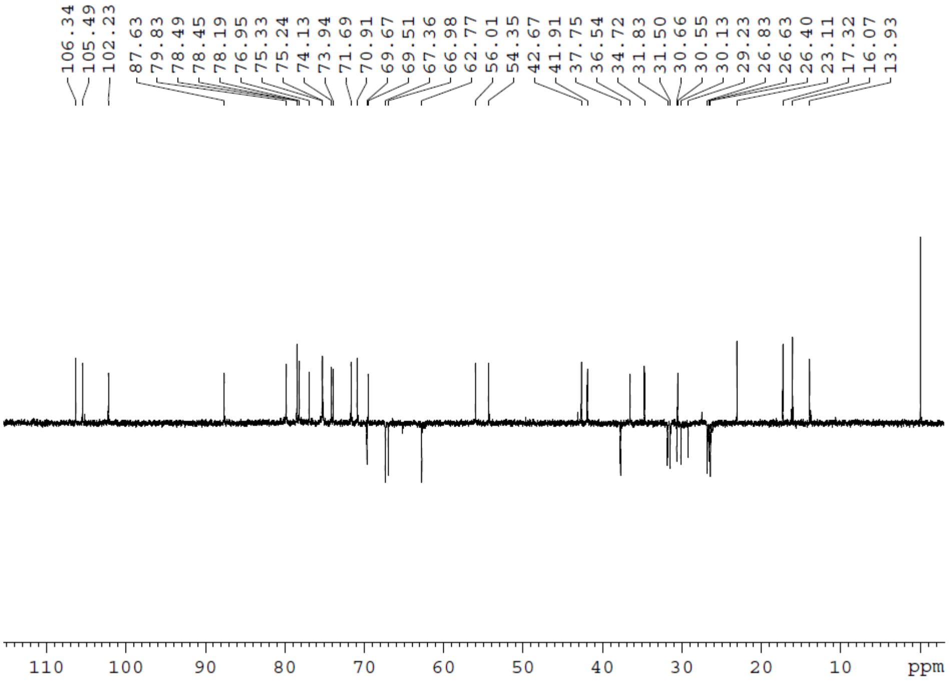

Figure S48. The DEPT 135 (C_5D_5N) spectrum of compound **38**

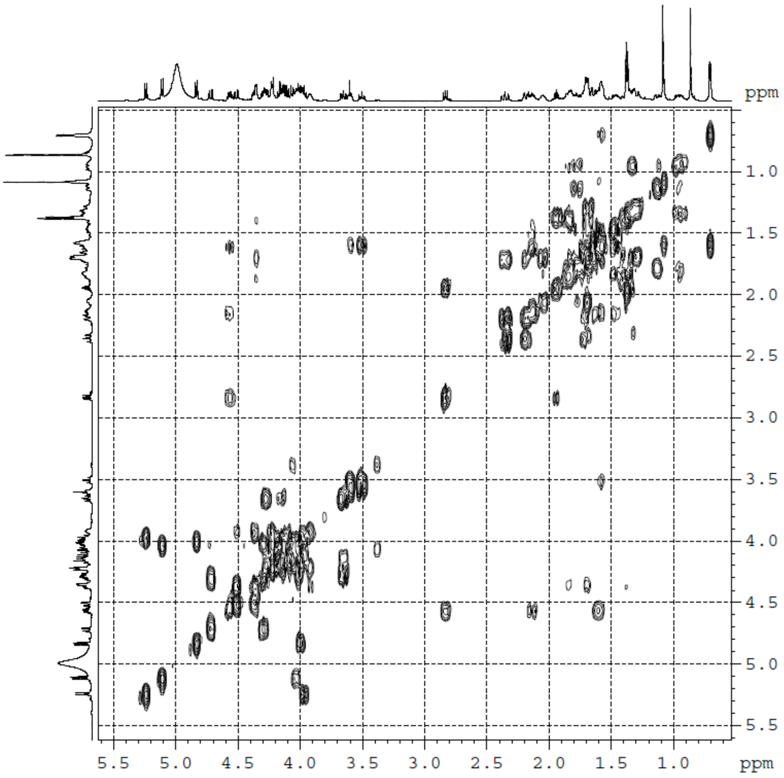

Figure S49. The ^1H ^1H COSY (C_5D_5N) spectrum of compound **38**

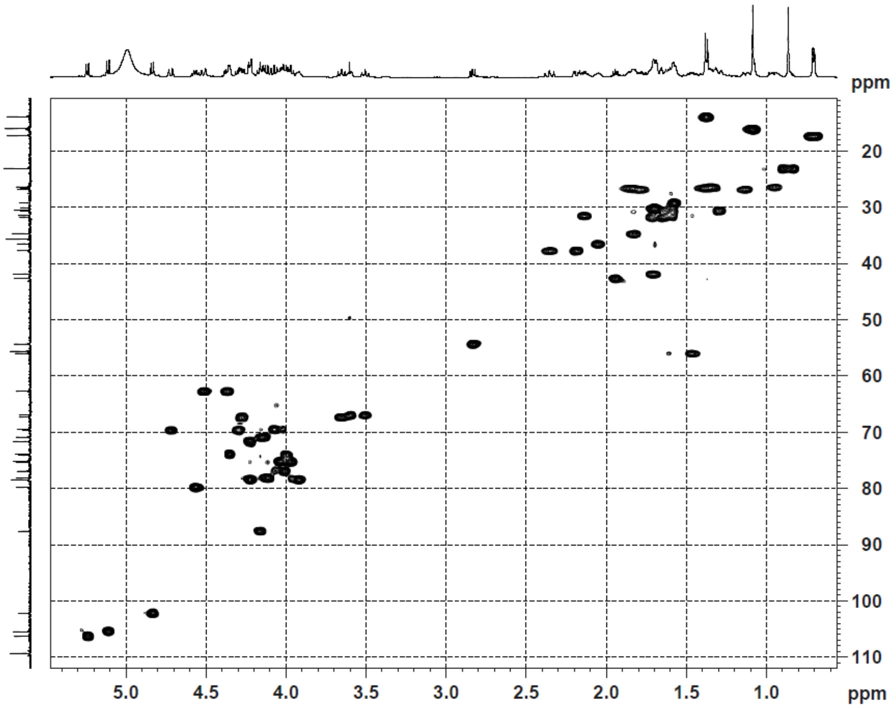

Figure S50. The HSQC (C_5D_5N) spectrum of compound **38**

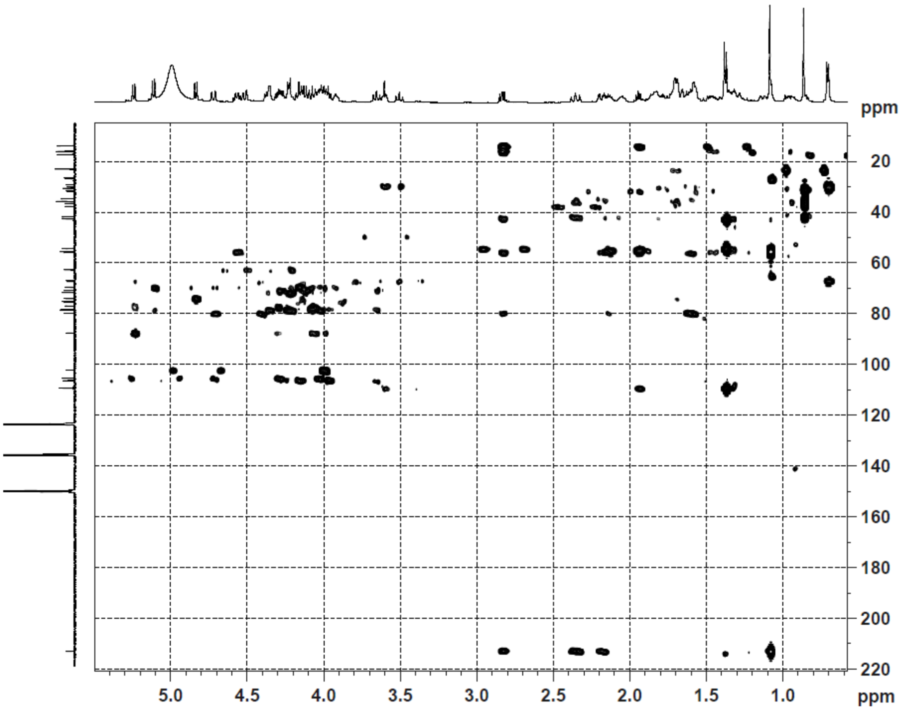

Figure S51. The HMBC (C_5D_5N) spectrum of compound **38**

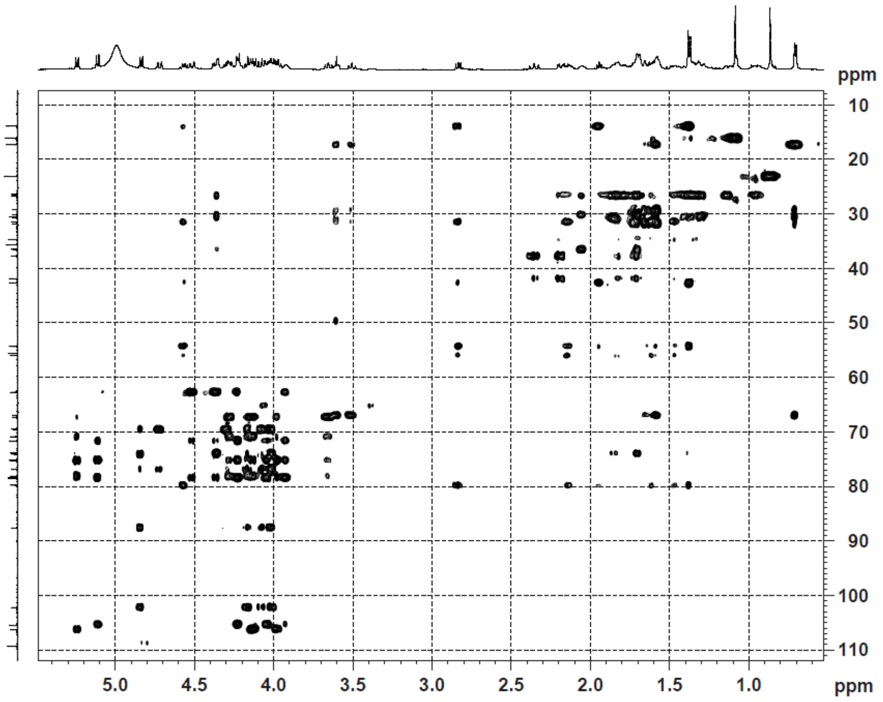

Figure S52. The HSQC-TOCSY (C_5D_5N) spectrum of compound **38**

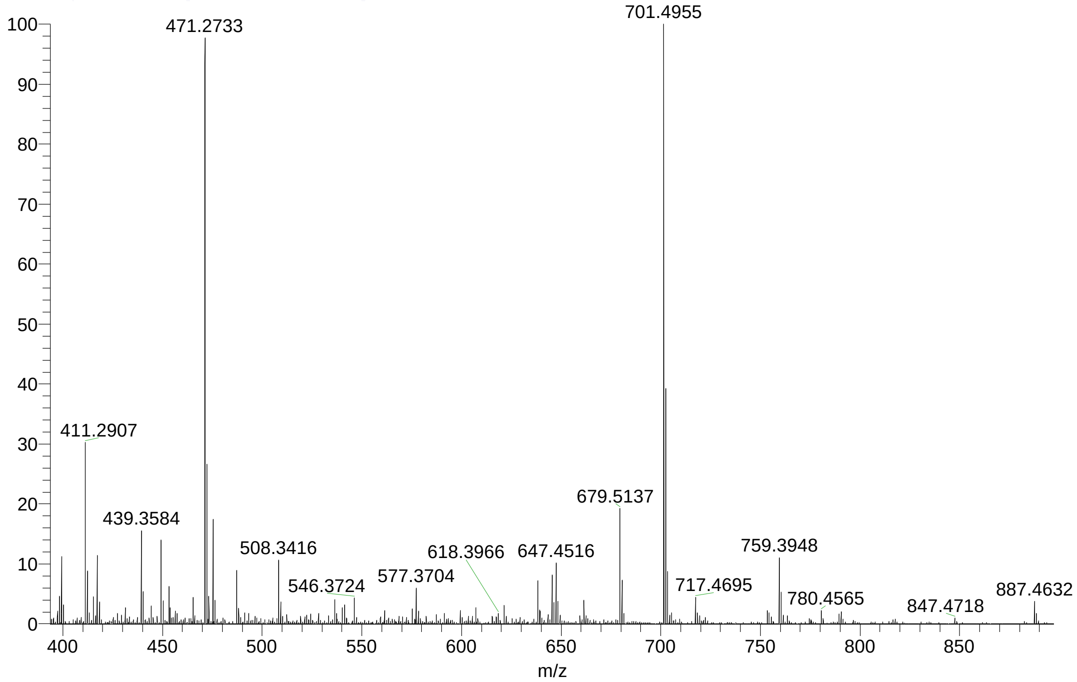

Figure S53. The ESI-Q-Orbitrap MS spectrum of compound **38**

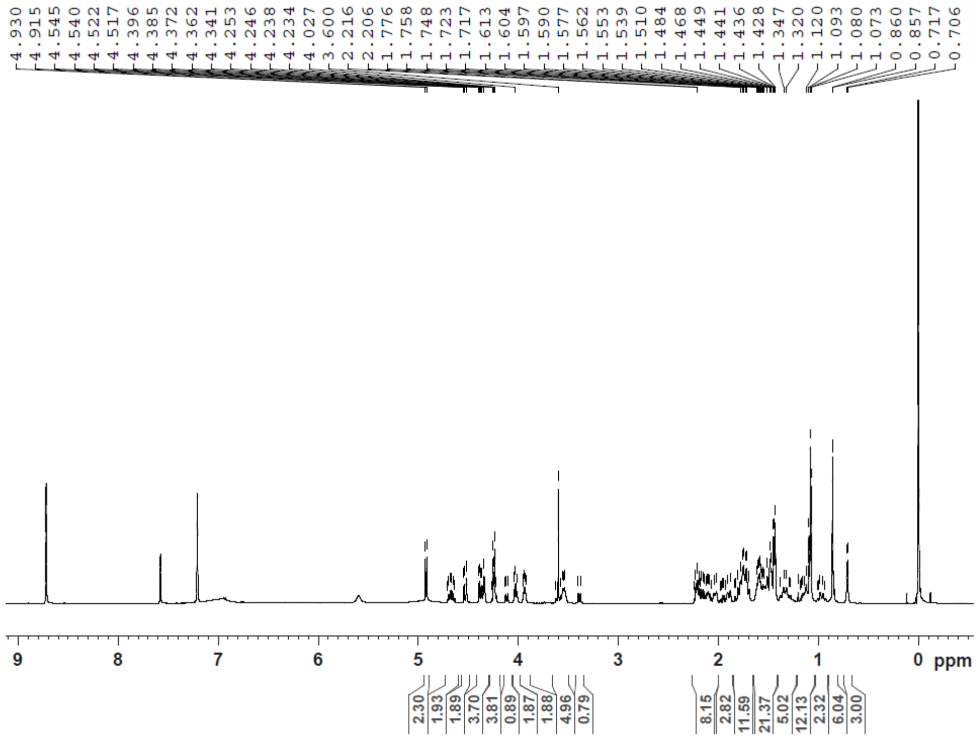

Figure S54. ^{1}H NMR (500MHz, C_5D_5N) spectrum of compound **40/41**

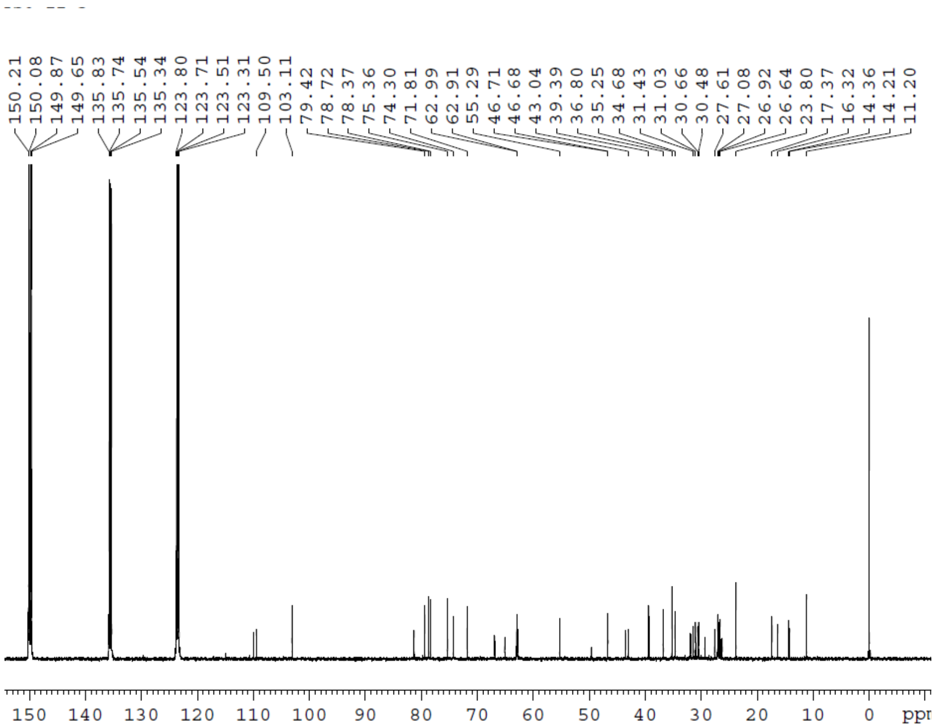

Figure S55. ^{13}C NMR (125MHz, C_5D_5N) spectrum of compound **40/41**

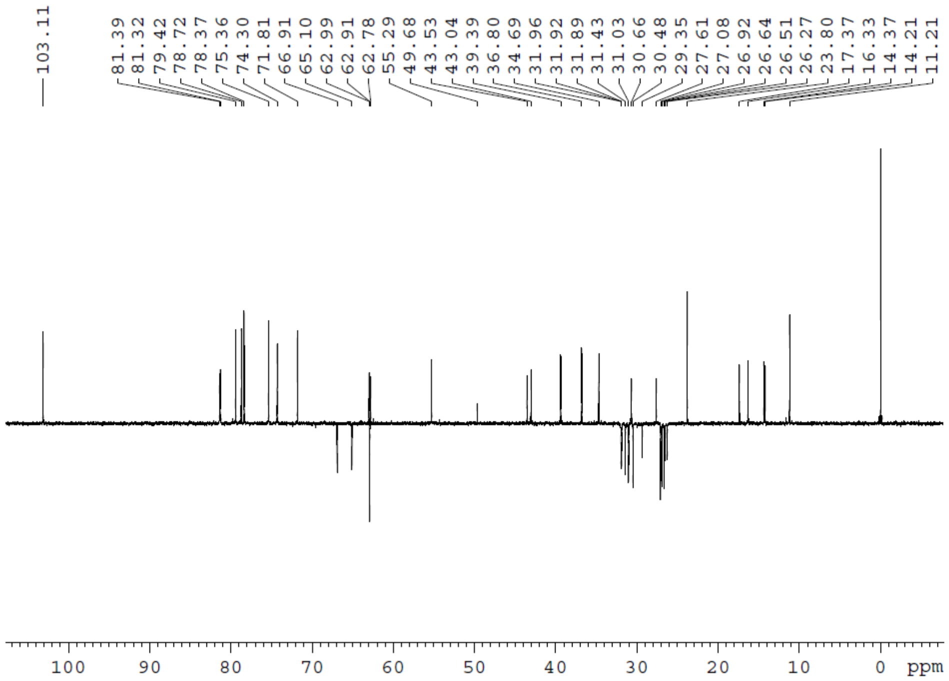

Figure S56. The DEPT 135 (C_5D_5N) spectrum of compound **40/41**

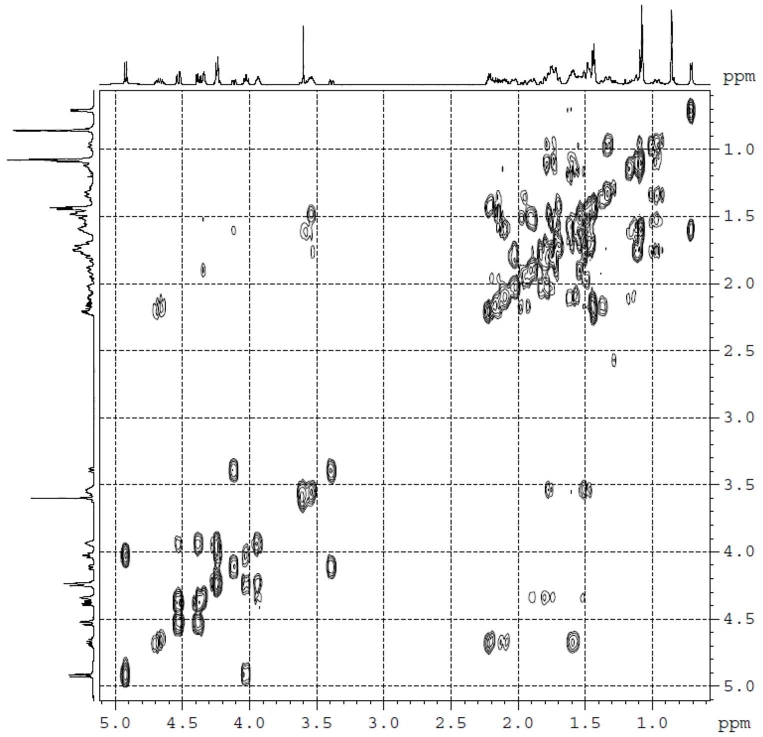

Figure S57. The ^1H ^1H COSY (C_5D_5N) spectrum of compound **40/41**

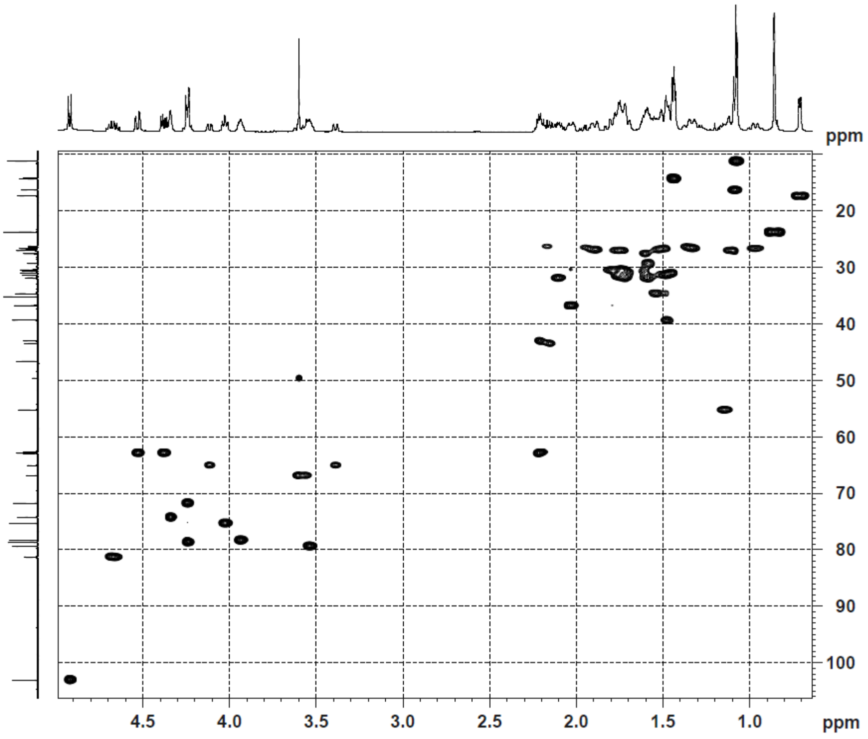

Figure S58. The HSQC (C_5D_5N) spectrum of compound **40/41**

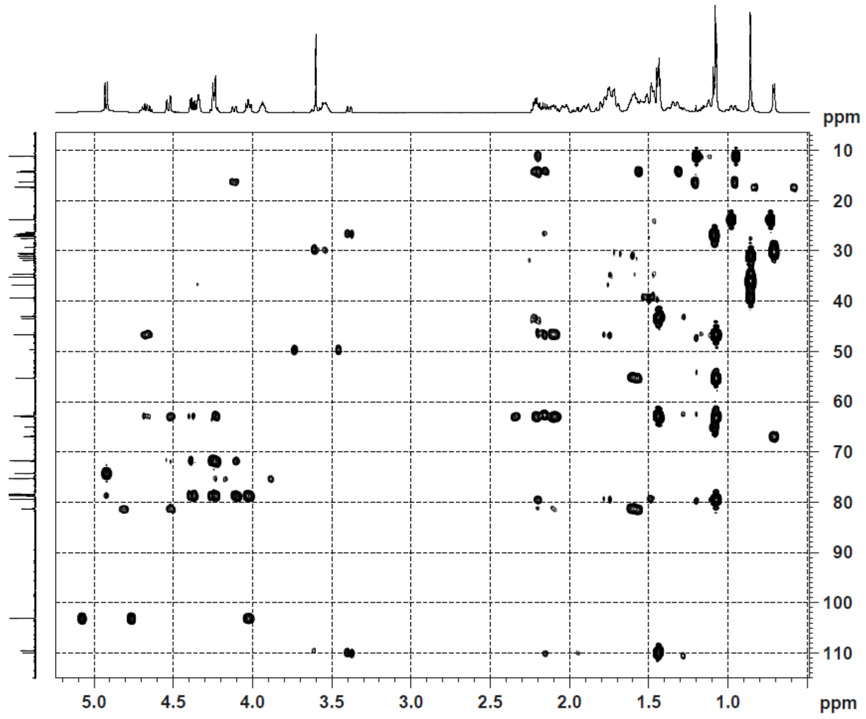

Figure S59. The HMBC (C_5D_5N) spectrum of compound **40/41**

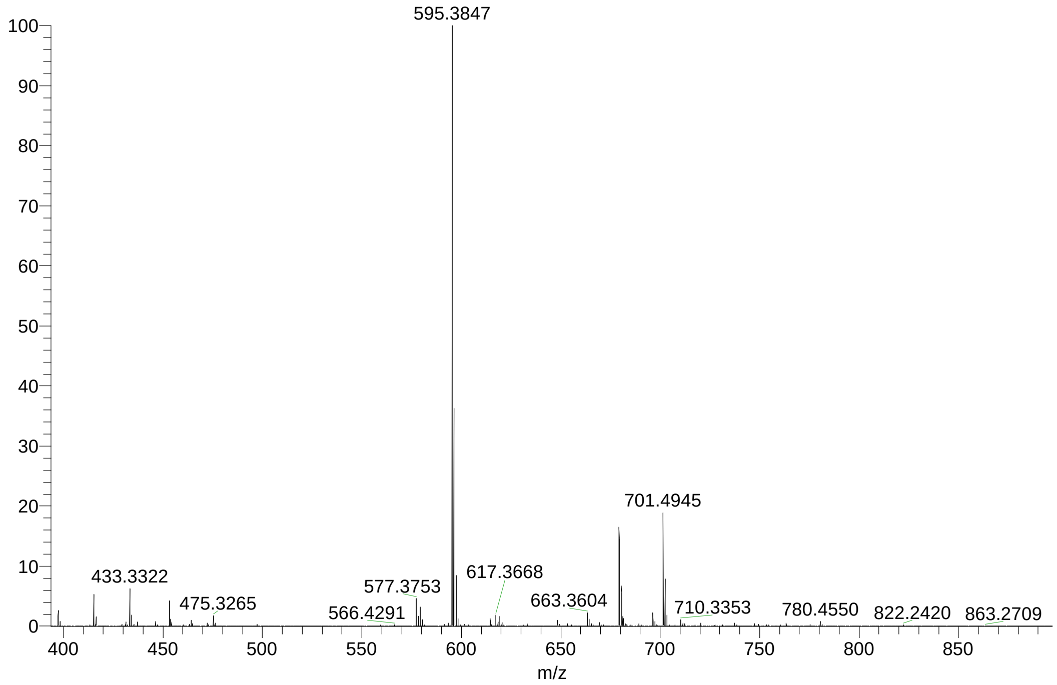

Figure S60. The ESI-Q-Orbitrap MS spectrum of compound **40/41**

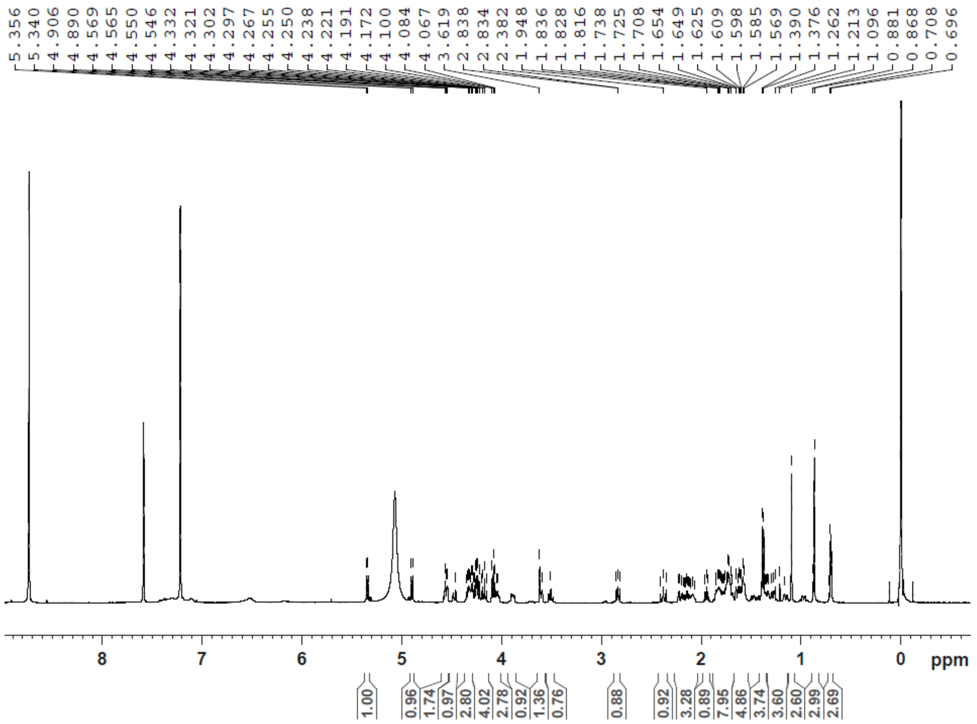

Figure S61. ^{1}H NMR (500MHz, C_5D_5N) spectrum of compound **51**

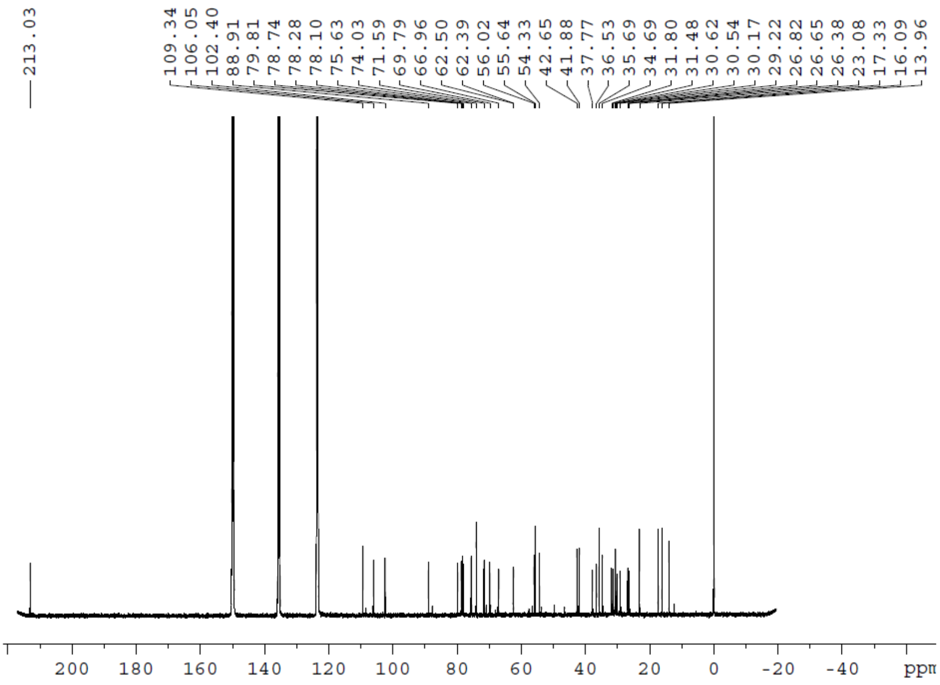

Figure S62. ^{13}C NMR (125MHz, C_5D_5N) spectrum of compound **51**

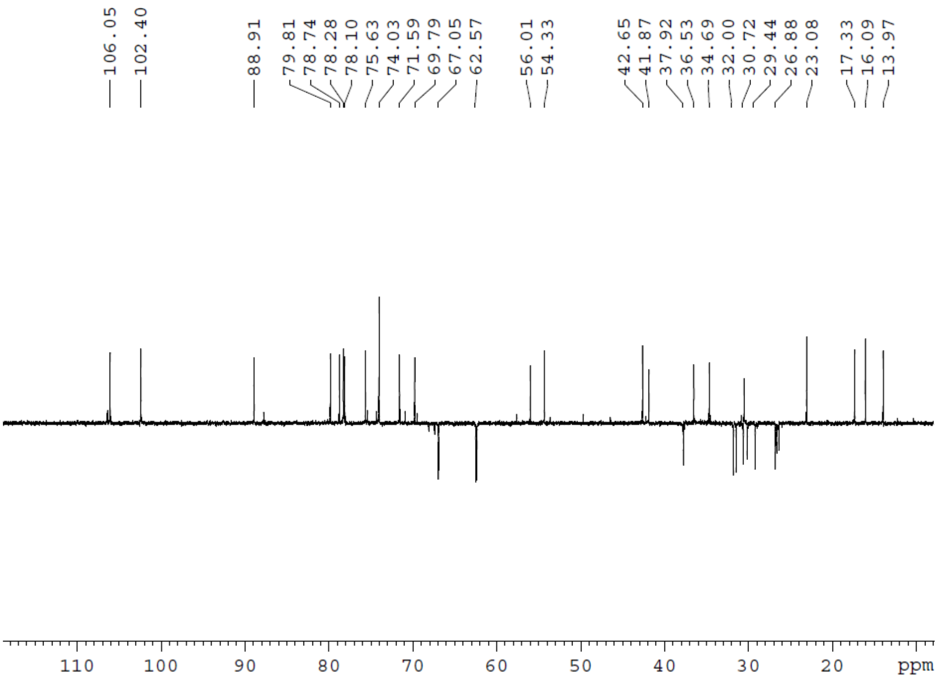

Figure S63. The DEPT 135 (C_5D_5N) spectrum of compound **51**

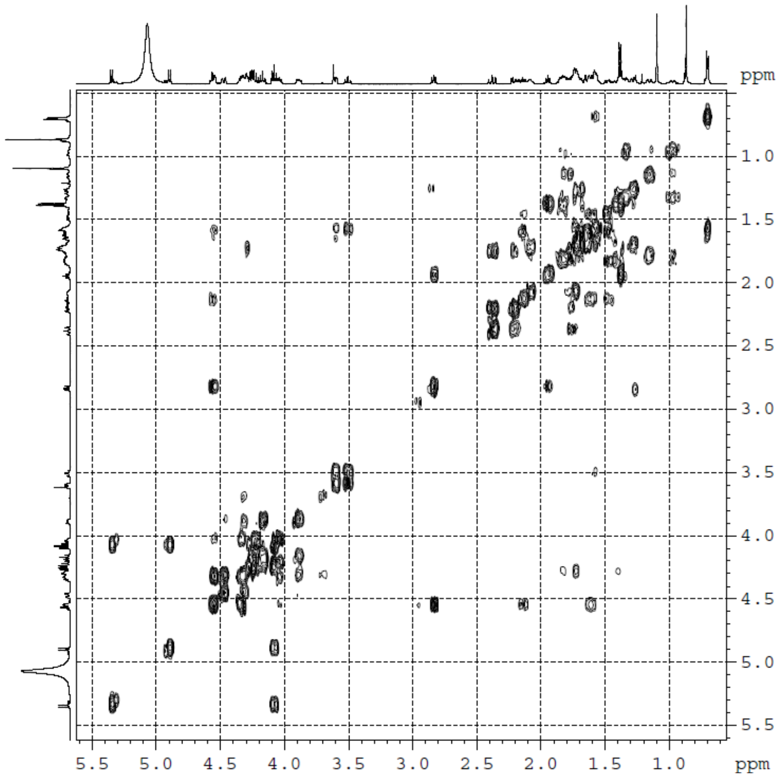

Figure S64. The ^1H ^1H COSY (C_5D_5N) spectrum of compound **51**

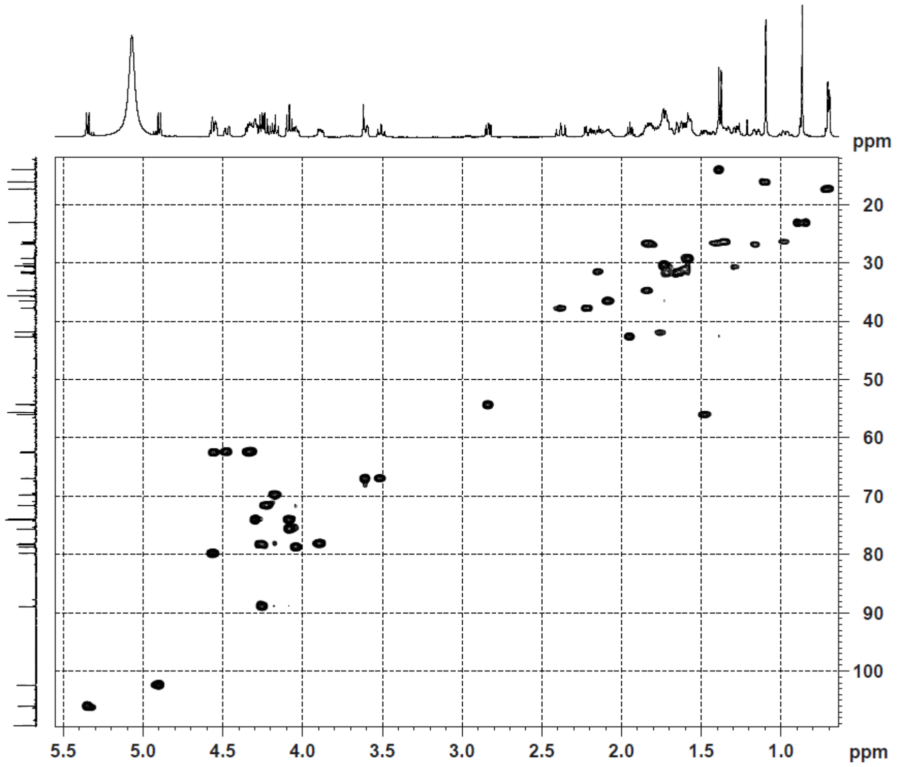

Figure S65. The HSQC (C_5D_5N) spectrum of compound **51**

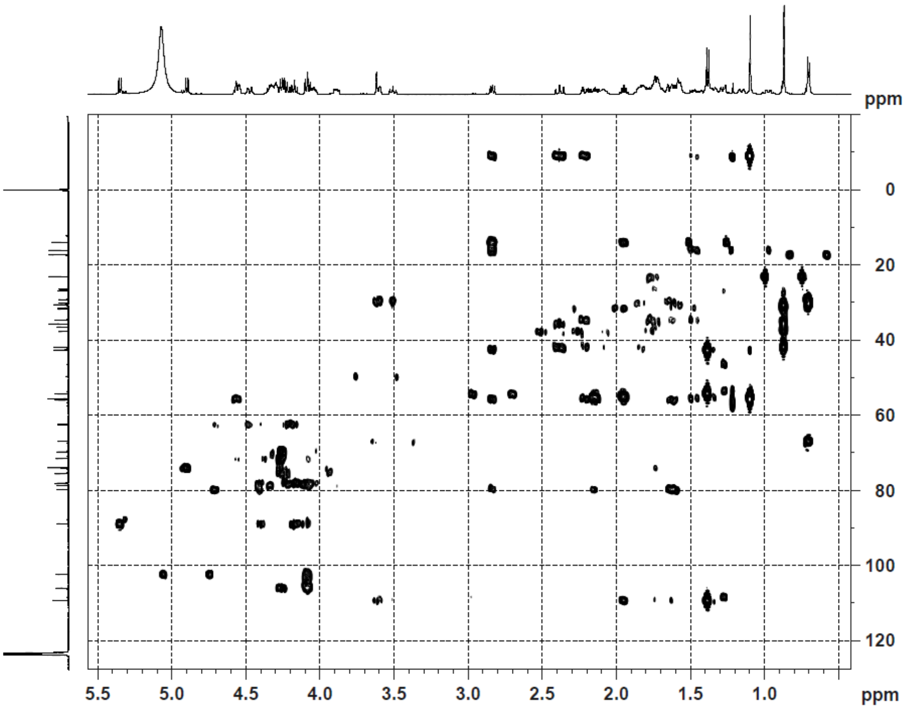

Figure S66. The HMBC (C_5D_5N) spectrum of compound **51**

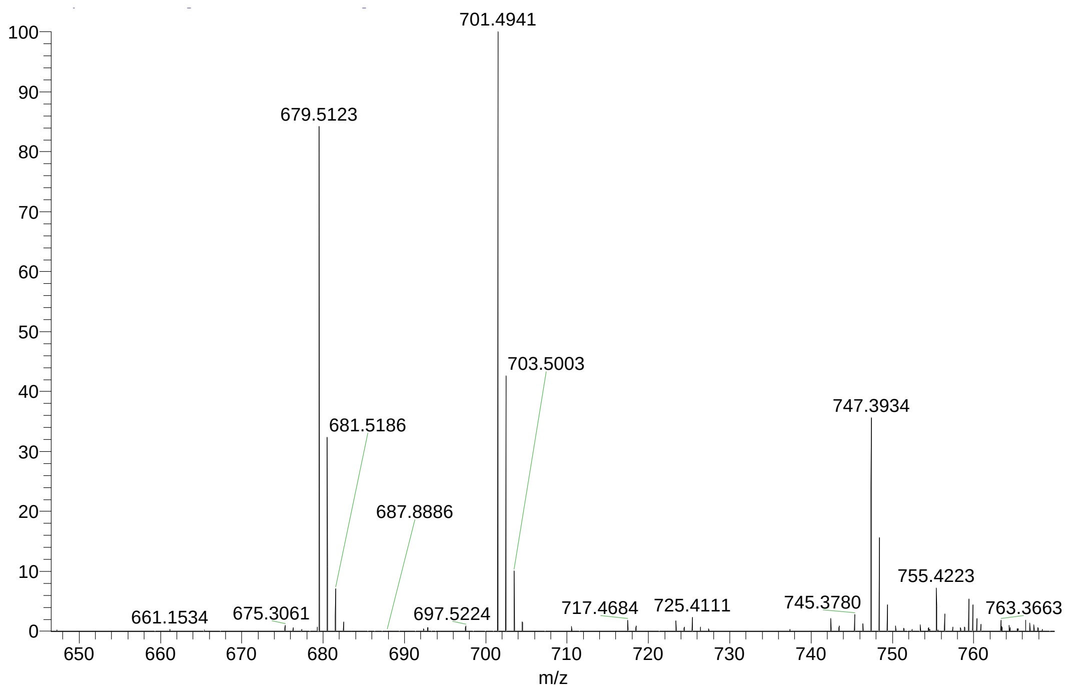

Figure S67. The ESI-Q-Orbitrap MS spectrum of compound **51**

Table S1. ^{1}H and ^{13}C NMR data for compound **10** in C_5D_5N

No.	δ_C	δ_H (*J* in Hz)	No.	δ_C	δ_H (*J* in Hz)
1	39.8	1.74 (m, overlapped)	22	109.3	—
		1.96 (m, overlapped)	23	31.8	1.65 (m)
2	66.8	3.75 (m, overlapped)			1.71 (m, overlapped)
3	79.8	4.40 (m, overlapped)	24	29.2	1.59 (m, overlapped)
4	30.7	1.82 (m, overlapped)	25	30.5	1.58 (m, overlapped)
		1.98 (m, overlapped)	26	67.0	3.50 (dd, 10.5, 10.5)
5	35.5	2.24 (m)			3.59 (m, overlapped)
6	26.2	1.30 (m, overlapped)	27	17.3	0.71 (d, 6.0)
		1.78 (m)	1'	101.7	4.98 (d, 7.5)
7	26.5	0.92 (m)	2'	77.3	4.87 (dd, 7.5, 9.5)
		1.30 (m, overlapped)	3'	84.2	4.30 (dd, 2.5, 9.5)
8	34.8	1.80 (m, overlapped)	4'	69.8	4.75 (br. d, *ca*. 3)
9	43.0	1.71 (m, overlapped)	5'	76.7	4.10 (m, overlapped)
10	37.5	—	6'	61.9	4.39 (m, overlapped)
11	37.9	2.35 (dd, 5.5, 14.0)	1''	104.4	5.57 (d, 7.5)
		2.40 (dd, 14.0, 14.0)	2''	76.4	4.03 (dd, 7.5, 9.5)
12	212.7	—	3''	78.7	4.20 (dd, 9.5, 9.5)
13	55.6	—	4''	72.8	4.08 (dd, 9.5, 9.5)
14	55.8	1.44 (m)	5''	77.9	3.76 (m, overlapped)
15	31.5	1.59 (m, overlapped)	6''	63.4	4.28 (dd, 6.5, 11.0)
		2.12 (ddd, 5.5, 7.0, 12.5)			4.48 (dd, 2.0, 11.0)
16	79.9	4.53 (q like, *ca*. 9)	1'''	106.2	5.23 (d, 8.0)
17	54.3	2.81 (dd, 7.0, 9.0)	2'''	75.2	3.94 (dd, 8.0, 9.0)
18	16.0	1.08 (s)	3'''	78.5	4.11 (m, overlapped)
19	23.0	0.98 (s)	4'''	71.1	4.13 (m, overlapped)
20	42.7	1.93 (quin, 7.0)	5'''	67.2	3.59 (m, overlapped)
21	13.9	1.35 (d, 7.0)			4.19 (dd, 4.5, 10.0)

Table S2a. ^{1}H and ^{13}C NMR data of C-1–15, 1'–6', 1''–6'', and 1'''–6''' for compound **14/15** in C_5D_5N

No.	δ_C	δ_H (*J* in Hz)	No.	δ_C	δ_H (*J* in Hz)
1	31.0	1.57 (m, overlapped)	1'	102.3	4.90 (d, 8.0)
		1.76 (m, overlapped)	2'	80.2	4.39 (dd, 8.0, 8.0)
2	26.7	1.47, 1.89 (both m)	3'	86.9	4.25 (m, overlapped)
3	75.7	4.30 (m)	4'	69.6	4.04 (dd, 9.0, 9.0)
4	30.9	1.73 (m, overlapped)	5'	77.9	3.80 (m)
		1.83 (m)	6'	62.4	4.26 (m, overlapped)
5	36.8	2.17 (m)			4.45 (dd, 2.0, 12.0)
6	27.1	1.19 (m)	1''	104.3	5.65 (d, 8.0)
		1.77 (m, overlapped)	2''	76.6	4.08 (m, overlapped)
7	26.7	0.94, 1.30 (both m)	3''	78.2	4.28 (m, overlapped)
8	34.7	1.52 (m)	4''	72.4	4.22 (dd, 9.0, 9.0)
9	39.4	1.44 (m, overlapped)	5''	78.3	3.96 (m)
10	35.3	—	6''	63.3	4.44 (m, overlapped)
11	31.4	1.49 (m, overlapped)			4.55 (dd, 3.0, 11.5)
		1.76 (m, overlapped)	1'''	105.2	5.29 (d, 7.5)
12	79.4	3.53 (m)	2'''	75.1	3.97 (dd, 7.5, 8.5)
13	46.7	—	3'''	78.6	4.11 (m, overlapped)
14	55.3	1.12 (m)	4'''	70.8	4.12 (m)
15	31.9	1.60 (m, overlapped)	5'''	67.4	3.67 (dd, 10.0, 10.0)
		2.10 (m, overlapped)			4.24 (m, overlapped)

Table S2b. ^{1}H and ^{13}C NMR data of C-16–27 for compound **14/15** in C_5D_5N

	25*R*		25*S*	
No.	δ_C	δ_H (*J* in Hz)	δ_C	δ_H (*J* in Hz)
16	81.4	4.70 (q like, *ca*. 8)	81.4	4.67 (q like, *ca*. 8)
17	63.0	2.23 (m, overlapped)	62.8	2.21 (m, overlapped)
18	11.2	1.08 (s)	11.2	1.07 (s)
19	23.9	0.96 (s)	23.9	0.96 (s)
20	43.1	2.22 (m, overlapped)	43.5	2.16 (m, overlapped)
21	14.4	1.45 (d, 6.5)	14.2	1.44 (d, 6.5)
22	109.5	—	110.0	—
23	32.0	1.60 (m, overlapped)	26.5	1.39 (m, overlapped)
		1.73 (m, overlapped)		1.94 (m)
24	29.4	1.60 (m, overlapped)	26.3	1.39 (m, overlapped)
				2.17 (m)
25	30.7	1.61 (m, overlapped)	27.6	1.60 (m, overlapped)
26	66.9	3.57 (dd, 11.5, 11.5)	65.1	3.39 (br. d, *ca*. 11)
		3.62 (m, overlapped)		4.13 (m, overlapped)
27	17.4	0.72 (d, 5.5)	16.3	1.09 (d, 7.5)

Table S3. ^1H and ^{13}C NMR data for compound **27** in C_5D_5N

No.	δ_C	δ_H (*J* in Hz)	No.	δ_C	δ_H (*J* in Hz)
1	30.1	1.27 (m)	23	31.9	1.66 (m)
		1.94 (m, overlapped)			1.72 (m, overlapped)
2	26.7	1.35 (m)	24	29.3	1.58 (m, overlapped)
		1.74 (m, overlapped)	25	30.6	1.59 (m, overlapped)
3	74.3	4.21 (m)	26	67.0	3.51 (dd, 10.5, 10.5)
4	30.3	1.71 (m, overlapped)			3.60 (dd, 3.0, 10.5)
5	35.8	2.34 (m)	27	17.3	0.71 (d, 5.5)
6	26.8	1.13 (m)	1'	100.5	4.81 (d, 7.0)
		1.74 (m, overlapped)	2'	78.7	4.24 (dd, 7.0, 8.5)
7	26.5	0.94, 1.31 (both m)	3'	88.7	4.05 (dd, 8.5, 9.0)
8	34.8	1.82 (m)	4'	69.6	3.99 (m, overlapped)
9	42.0	1.74 (m, overlapped)	5'	78.0	3.72 (m)
10	35.8	—	6'	62.2	4.27 (dd, 5.0, 12.0)
11	37.8	2.21 (dd, 4.5, 14.0)			4.42 (dd, 2.0, 12.0)
		2.38 (dd, 14.0, 14.0)	1''	104.0	5.53 (d, 8.0)
12	213.1	—	2''	76.2	4.00 (m, overlapped)
13	55.7	—	3''	78.3	4.28 (dd, 8.5, 9.0)
14	56.1	1.46 (m)	4''	72.9	4.12 (dd, 9.0, 9.5)
15	31.5	1.59 (m, overlapped)	5''	78.2	3.96 (m, overlapped)
		2.13 (m)	6''	63.6	4.38 (dd, 6.0, 11.5)
16	79.8	4.55 (q like, *ca.* 9)			4.59 (dd, 3.0, 11.5)
17	54.4	2.83 (dd, 6.5, 8.5)	1'''	111.5	6.07 (d, 4.5)
18	16.1	1.10 (s)	2'''	77.3	4.80 (d, 4.5)
19	23.1	1.01 (s)	3'''	80.2	—
20	42.7	1.95 (m, overlapped)	4'''	74.8	4.29, 4.71 (both d, 9.5)
21	14.0	1.38 (d, 7.0)	5'''	64.1	4.09 (m)
22	109.3	—			

Table S4a. ^{1}H and ^{13}C NMR data of C-1–15, 1'–6', and 1''–5'' for compound **34/36** in C_5D_5N

No.	δ_C	δ_H (J in Hz)	No.	δ_C	δ_H (J in Hz)
1	31.0	1.46 (m)	13	46.7	—
		1.74 (m, overlapped)	14	55.3	1.15 (m, overlapped)
2	26.9	1.50, 1.84 (both m)	15	31.9	1.60 (m, overlapped)
3	74.4	4.32 (m, overlapped)			2.11 (m, overlapped)
4	30.4	1.74 (m, overlapped)	1'	102.6	4.90 (d, 8.0)
		1.81 (m, overlapped)	2'	74.2	4.05 (dd, 8.0, 8.5)
5	36.8	2.03 (m)	3'	87.8	4.22 (dd, 8.5, 9.0)
6	27.1	1.14 (m)	4'	69.6	4.15 (dd, 9.0, 9.5)
		1.79 (m, overlapped)	5'	78.1	3.89 (m)
7	26.7	0.98 (m)	6'	62.5	4.30 (dd, 5.0, 12.0)
		1.35 (m, overlapped)			4.47 (dd, 2.0, 12.0)
8	34.7	1.54 (m)	1''	106.3	5.27 (d, 7.5)
9	39.4	1.48 (m, overlapped)	2''	75.3	4.01 (dd, 7.5, 8.5)
10	35.3	—	3''	78.2	4.12 (dd, 8.5, 8.5)
11	31.4	1.49 (m, overlapped)	4''	70.9	4.14 (m)
		1.77 (m, overlapped)	5''	67.4	3.68 (dd, 9.5, 11.0)
12	79.4	3.54 (m)			4.31 (m, overlapped)

Table S4b. ^{1}H and ^{13}C NMR data of C-16–27 for compound **34/36** in C_5D_5N

	**25*R***		**25*S***	
**No.**	δ_C	δ_H (J in Hz)	δ_C	δ_H (J in Hz)
16	81.3	4.68 (q like, *ca*. 8)	81.4	4.65 (q like, *ca*. 8)
17	63.0	2.22 (m, overlapped)	62.8	2.20 (m, overlapped)
18	11.2	1.08 (s)	11.2	1.08 (s)
19	23.8	0.88 (s)	23.8	0.88 (s)
20	43.1	2.21 (m, overlapped)	43.5	2.16 (m, overlapped)
21	14.4	1.44 (d, 7.0)	14.2	1.43 (d, 7.0)
22	109.5	—	110.0	—
23	32.0	1.61 (m, overlapped)	26.5	1.36 (m, overlapped)
		1.72 (m)		1.95 (m)
24	29.4	1.58 (m, overlapped)	26.3	1.37 (m, overlapped)
				2.17 (m, overlapped)
25	30.7	1.59 (m, overlapped)	27.6	1.60 (m, overlapped)
26	66.9	3.55 (dd, 10.0, 10.0)	65.1	3.39 (br. d, *ca*. 11)
		3.60 (m, overlapped)		4.11 (m, overlapped)
27	17.4	0.71 (d, 5.5)	16.3	1.09 (d, 5.0)

Table S5. ^{1}H and ^{13}C NMR data for compound **38** in C_5D_5N

No.	δ_C	δ_H (J in Hz)	No.	δ_C	δ_H (J in Hz)
1	30.7	1.30 (m)	23	31.8	1.65 (m)
		1.70 (m, overlapped)			1.71 (m, overlapped)
2	26.6	1.36 (m, overlapped)	24	29.2	1.58 (m, overlapped)
		1.83 (m, overlapped)	25	30.6	1.59 (m, overlapped)
3	73.9	4.35 (m, overlapped)	26	67.0	3.51 (dd, 10.5, 10.5)
4	30.1	1.71 (m, overlapped)			3.60 (dd, 3.5, 10.5)
5	36.5	2.04 (m)	27	17.3	0.71 (d, 5.5)
6	26.8	1.12, 1.79 (both m)	1'	102.2	4.83 (d, 7.5)
7	26.4	0.95 (m)	2'	74.1	4.00 (dd, 7.5, 9.0)
		1.34 (m, overlapped)	3'	87.6	4.16 (dd, 9.0, 9.0)
8	34.7	1.83 (m, overlapped)	4'	69.5	4.08 (dd, 9.0, 9.0)
9	41.9	1.71 (m, overlapped)	5'	77.0	4.01 (m)
10	35.7	—	6'	69.7	4.30 (dd, 5.5, 12.0)
11	37.7	2.19 (dd, 5.0, 13.5)			4.72 (dd, 1.5, 12.0)
		2.36 (dd, 13.5, 13.5)	1''	106.3	5.24 (d, 7.5)
12	213.0	—	2''	75.3	3.97 (dd, 7.5, 8.0)
13	55.6	—	3''	78.2	4.11 (dd, 8.0, 8.0)
14	56.0	1.46 (m)	4''	70.9	4.15 (m)
15	31.5	1.59 (m, overlapped)	5''	67.4	3.65 (dd, 10.0, 10.0)
		2.13 (m)			4.28 (dd, 5.0, 10.0)
16	79.8	4.57 (q like, *ca*. 7)	1'''	105.5	5.11 (d, 8.0)
17	54.4	2.83 (dd, 6.5, 8.5)	2'''	75.2	4.04 (m, overlapped)
18	16.1	1.09 (s)	3'''	78.5	4.23 (m, overlapped)
19	23.1	0.87 (s)	4'''	71.7	4.23 (m, overlapped)
20	42.7	1.94 (quin, 7.0)	5'''	78.5	3.92 (m)
21	13.9	1.38 (d, 7.0)	6'''	62.8	4.37 (dd, 5.5, 11.5)
22	109.4	—			4.52 (dd, 2.0, 11.5)

Table S6a. ^{1}H and ^{13}C NMR data of C-1–15 and C-1'–6' for compound **40/41** in C_5D_5N

**No.**	δ_C	δ_H (*J* in Hz)	**No.**	δ_C	δ_H (*J* in Hz)
1	31.0	1.45 (m)	11	31.4	1.49 (m, overlapped)
		1.76 (m, overlapped)			1.77 (m, overlapped)
2	26.9	1.52, 1.89 (both m)	12	79.4	3.54 (m)
3	74.3	4.34 (m)	13	46.7	—
4	30.5	1.73 (m, overlapped)	14	55.3	1.15 (m)
		1.80 (m)	15	31.9	1.60 (m, overlapped)
5	36.8	2.03 (m)			2.11 (m, overlapped)
6	27.1	1.11 (m)	1'	103.1	4.92 (d, 7.5)
		1.75 (m, overlapped)	2'	75.4	4.03 (dd, 7.5, 9.0)
7	26.6	0.97 (m)	3'	78.7	4.24 (m, overlapped)
		1.35 (m, overlapped)	4'	71.8	4.24 (m, overlapped)
8	34.7	1.54 (m)	5'	78.4	3.94 (m)
9	39.4	1.48 (m, overlapped)	6'	62.9	4.38 (dd, 5.0, 12.0)
10	35.3	—			4.53 (dd, 2.5, 12.0)

Table S6b. ^{1}H and ^{13}C NMR data of C-16–27 for compound **40/41** in C_5D_5N

	**25*R***		**25*S***	
**No.**	δ_C	δ_H (*J* in Hz)	δ_C	δ_H (*J* in Hz)
16	81.3	4.69 (q like, *ca.* 8)	81.4	4.66 (q like, *ca.* 8)
17	63.0	2.22 (m, overlapped)	62.8	2.20 (m, overlapped)
18	11.2	1.08 (s)	11.2	1.07 (s)
19	23.8	0.86 (s)	23.8	0.84 (s)
20	43.0	2.21 (m, overlapped)	43.5	2.15 (m, overlapped)
21	14.4	1.44 (d, 6.5)	14.2	1.43 (d, 6.5)
22	109.5	—	110.0	—
23	32.0	1.62 (m, overlapped)	26.5	1.36 (m, overlapped)
		1.71 (m)		1.94 (m)
24	29.4	1.59 (m, overlapped)	26.1	1.36 (m, overlapped)
				2.17 (m)
25	30.7	1.60 (m, overlapped)	27.6	1.60 (m, overlapped)
26	66.9	3.56 (dd, 10.5, 10.5)	65.1	3.39 (br. d, *ca.* 11)
		3.60 (dd, 3.0, 10.5)		4.12 (dd, 2.5, 10.5)
27	17.4	0.71 (d, 5.5)	16.3	1.09 (d, 6.5)

Table S7. ^1H and ^{13}C NMR data for compound **51** in C_5D_5N

No.	δ_C	δ_H (*J* in Hz)	No.	δ_C	δ_H (*J* in Hz)
1	30.6	1.28 (m)	21	14.0	1.38 (d, 7.0)
		1.70 (m, overlapped)	22	109.3	—
2	26.7	1.43 (m)	23	31.8	1.65 (m, overlapped)
		1.82 (m, overlapped)			1.72 (m, overlapped)
3	74.0	4.30 (m)	24	29.2	1.58 (m, overlapped)
4	30.2	1.73 (m, overlapped)	25	30.5	1.57 (m, overlapped)
5	36.5	2.09 (m)	26	67.0	3.51 (dd, 10.5, 10.5)
6	26.8	1.15, 1.79 (both m)			3.61 (dd, 3.0, 10.5)
7	26.4	0.98, 1.35 (both m)	27	17.3	0.70 (d, 6.0)
8	34.7	1.83 (m, overlapped)	1'	102.4	4.89 (d, 8.0)
9	41.9	1.75 (m, overlapped)	2''	74.0	4.08 (dd, 8.0, 8.5)
10	35.7	—	3'	88.9	4.25 (dd, 8.5, 8.5)
11	37.8	2.21 (dd, 4.5, 13.5)	4'	69.8	4.17 (dd, 8.5, 9.0)
		2.38 (dd, 13.5, 13.5)	5'	78.1	3.89 (m)
12	213.0	—	6'	62.4	4.32 (m, overlapped)
13	55.6	—			4.47 (br. d, *ca.* 12)
14	56.0	1.47 (m)	1''	106.1	5.35 (d, 8.0)
15	31.5	1.60 (m, overlapped)	2'	75.6	4.08 (dd, 8.0, 8.5)
		2.15 (ddd, 5.5, 8.0, 11.5)	3''	78.3	4.27 (dd, 8.5, 8.5)
16	79.8	4.54 (m, overlapped)	4''	71.6	4.22 (dd, 8.5, 8.5)
17	54.3	2.84 (dd, 6.5, 8.5)	5''	78.7	4.05 (m)
18	16.1	1.10 (s)	6''	62.5	4.34 (dd, 4.0, 12.0)
19	23.1	0.87 (s)			4.55 (m, overlapped)
20	42.7	1.95 (quin, 7.0)			

Table S8. The characteristic types of substituted glycosyl groups

Glycosyl types	The glycosyl directly linked with aglycon (DG)	2'-DG	3'-DG	6'-DG	Structure
Disaccharide glycoside	Glc	Glc	Glc/Xyl	—	A_2–A_5
	Gal	Glc	Glc/Xyl	—	
Trisaccharide glycoside	Glc	Glc	Glc/Xyl	Glc	A_6–A_9
	Gal	Glc	Glc/Xyl	—	

Table S9. The characteristic fragment ions of seven aglycone moieties

Aglycone	*m*/*z*
**YS-1**	415, 397, 379, 285, 273, 255
**YS-2**	431, 413, 395, 377, 301, 289, 283, 271, 253
**YS-3**	429, 411, 393, 375, 317, 299, 287, 281, 269, 251
**YS-4**	431, 413, 395, 377, 301, 289, 283, 271, 253
**YS-5**	445, 427, 409, 391, 333, 315, 297, 279
**YS-6**	417, 399, 381, 285, 273, 255
**YS-8**	431, 413, 395, 377, 317, 299, 287, 281, 269, 251

Table S10. The chromatographic elution order of spirostanol saponins from YSESs

Effect factor	compound	t_R (min)	Elution order
**C-12 substitution**	(YS-1)-3-O*A₁* (peak **100**)	57.13 min	12-*β*-OH, 12-C=O, 12-H_2
	(YS-2)-3-OA_1 (peak **35**)	31.79 min	
	(YS-3)-3-OA_1 (peak **52**)	38.27 min	
**C-25 substitution**	(YS-1)-3-OA_1 (peak **100**)	57.13 min	$\Delta^{25(27)}$, 25-CH_3
	(25*S*)-(YS-6)-3-OA_1 (peak **104**)	58.57 min	
	(YS-3)-3-OA_1 (peak **52**)	38.27 min	
	(25*S*)-(YS-8)-3-OA_1 (peak **60**)	40.86 min	
**25-CH_3 stereochemistry**	(25*S*)-(YS-6)-3-OA_2 (peak **83**)	50.76 min	(25*S*)-CH_3, (25*R*)-CH_3
	(25*R*)-(YS-6)-3-OA_2 (peak **85**)	51.65 min	
	(25*S*)-(YS-6)-3-OA_3 (peak **84**)	51.46 min	
	(25*R*)-(YS-6)-3-OA_3 (peak **86**)	52.10 min	
**Substituted glycosyl**	(YS-3)-3-OA_9 (peak **100**)	24.76 min	Gal, Glc, Xyl
	(YS-3)-3-OA_8 (peak **17**)	26.12 min	
	(YS-1)-3-OA_8 (peak **66**)	44.37 min	
	(YS-1)-3-OA_6 (peak **72**)	46.88 min	
